# Encapsulation of *Monascus* Pigments Using Enzyme-Modified Yeast Protein–Polysaccharide Complex Pickering Emulsions to Increase Its Stability During Storage

**DOI:** 10.3390/foods14081366

**Published:** 2025-04-15

**Authors:** Ziyan Zhao, Jinling Zhao, Sirong Liu, Mengxuan Liu, Xiangquan Zeng, He Li, Yu Xi, Jian Li

**Affiliations:** 1 Key Laboratory of Geriatric Nutrition and Health, Ministry of Education, Beijing Technology and Business University, Beijing 100048, China; 18726926317@163.com (Z.Z.); 15110631956@163.com (J.Z.); 15223376402@163.com (S.L.); 15545957141@163.com (M.L.); lihe@btbu.edu.cn (H.L.); xiyu@btbu.edu.cn (Y.X.); 2 Key Laboratory of Green and Low-Carbon Processing Technology for Plant-Based Food of China National Light Industry Council, School of Food and Health, Beijing Technology and Business University, Beijing 100048, China; 3 Beijing Engineering and Technology Research Center of Food Additives, School of Food and Health, Beijing Technology and Business University, Beijing 100048, China

**Keywords:** enzyme-modified yeast protein, chitosan, xanthan gum, complex Pickering emulsions, stability

## Abstract

Yeast protein (YP) is rich in nutrients, but its emulsifying properties, especially emulsifying stability, still need to be improved. In this study, cationic polysaccharide chitosan (CS) and anionic polysaccharide xanthan gum (XG) were selected to enhance the emulsifying properties of protein emulsions. The preparation conditions of the emulsions were optimized by calculating particle size, zeta potential, emulsifying activity index, emulsifying stability index, and emulsifying capacity index. The optimized emulsions were characterized using confocal laser scanning microscopy, rheology, Raman spectroscopy, color difference analysis, and storage stability. The results showed that the stability of yeast protein/modified yeast protein–chitosan (YP/EYP–CS) emulsions was better at pH 5.5, with a protein:polysaccharide ratio of 1:1 and an oil phase addition of 40%, while the stability of yeast protein/modified yeast protein–xanthan gum (YP/EYP–XG) emulsions was better at pH 3.5, with a protein:polysaccharide ratio of 1:1 and an oil phase addition of 50%. Further analysis indicated that the emulsions with CS had smaller particle sizes and lower initial viscosities, but more hydrogen bonds and better encapsulation of *Monascus* pigment (MP), especially the EYP–CS emulsion (81.18%). In contrast, the emulsions with XG had uniform droplet sizes and high thermal stability and exhibited obvious shear thinning behavior with increasing shear rates. The network structure of the emulsions was mainly elastic, and the hydrophobic interaction was stronger. This study provides insights into the utilization of yeast protein in the food industry and the development of emulsification systems.

## 1. Introduction

*Monascus* pigments (MPs) are natural colorants produced through *Monascus* fermentation and have been widely utilized in the food industry for coloring meat products, beverages, and pastries. In addition to their coloring properties, MPs exhibit a range of health-promoting effects, including anti-diabetic, anti-inflammatory, anti-obesity, and anti-cancer activities [1,2]. However, the stability of MPs remains a critical limitation due to their inherent sensitivity to environmental stresses including thermal processing, extreme pH conditions, light exposure, and oxidative environments [3,4], which can lead to color loss and degradation, limiting their applications in food processing. Nowadays, different techniques have been developed to increase the stability of MPs, including ionic gelation [5], gel in oil in water (G/O/W) double emulsion [6], microgelation [7], emulsion package [8], and nanoparticles [9]. Among them, microencapsulation is one of the most common and effective techniques to improve the stability and shelf-life of MPs under different conditions [6]. Protein–polysaccharide complex-based emulsions exhibit superior stability [10]. Chitosan, a natural polyelectrolyte derived from chitin deacetylation, possesses excellent biocompatibility and biodegradability [11]. Xanthan gum, a heteropolysaccharide composed of repeating pentasaccharide units (glucose:mannose:glucuronic acid = 2:2:1), demonstrates unique shear thinning behavior and high viscosity. These polysaccharides typically enhance emulsion stability by forming extended networks that increase continuous phase viscosity, thereby retarding droplet coalescence. Notably, Pickering emulsions prepared using protein and polysaccharide complexes show high encapsulation efficiency, which could significantly increase the stability of MPs [12,13]. For example, heat-denatured whey protein and pectin modified by octenyl succinic anhydride were prepared into a complex by adding Ca^2+^ loaded with MPs. The complex carrier significantly enhanced the light and heat stabilities of MPs and exhibited excellent wettability [14].

As a kind of microbial protein obtained by wall breaking, separation, and purification of *Saccharomyces cerevisiae*, yeast protein (YP) has attracted increasing attention of researchers in recent years due to its short production cycle, high digestibility and nutritional value, as well as environmental friendliness [15,16]. YP contains more than 80% protein, with a utilization rate of approximately 96%. Studies have shown that its essential amino acid content surpasses that of soybean, wheat, and pea proteins, making its nutritional value align with the amino acid requirements for adults as outlined by the FAO. At present, YP has been applied in the production of bread, protein bars, sausages, chicken fillets, beef fillets, and other food products. Previously, a series of novel plant-based meat analogs were developed by combining YP and soybean isolate protein or konjac glucomannan using a high moisture extrusion technique [17]. In addition, YP has been successfully utilized as an animal fat substitute for emulsified sausages to improve the nutritional quality and reduce its fat content [18]. In recent years, some researchers have successfully fabricated novel Pickering emulsions using YP as wall materials to encapsulate a series of functional components (betaine, myricetin) to increase their physicochemical and biological properties. For instance, Hu et al. (2024) prepared novel emulsions of YP and chitooligosaccharide to encapsulate betaine and greatly increased its storage stability [19]. However, previous studies have shown that the emulsifying properties of YP are relatively poor, which limits its further application in the delivery of bioactive compounds in food systems [15,20]. Therefore, it is necessary to utilize effective methods to improve the emulsifying properties of YP to broaden its applications in the delivery of food functional components.

Physical, chemical, and enzymatic modifications have been effectively employed in previous studies to enhance the properties of proteins. Among these, enzymatic modification is the most commonly used due to its mild reaction conditions, ease of control, and minimal impact on the nutritional value of protein molecules [21]. Generally, enzymatic modification techniques include enzymatic hydrolysis and enzymatic cross-linking modification. After hydrolysis, an increase in the number of ionizable groups and exposure of the covered hydrophobic sheet can be observed, and the protein hydrolysates may have lower molecular weight and better biological activities than the original proteins [21]. According to the research of Lv et al. (2023) [22], the emulsifying activity of proteins significantly increases due to the exposure of hydrophobic amino acids after enzymatic hydrolysis. A series of hydrolases are applied in the modification of protein molecules, such as papain, alkaline protease, and neutral protease. Papain is capable of cleaving peptide bonds within hydrophobic regions, especially at the C-terminus of Lys and Arg residues. Alkaline protease, a serine endo-peptidase, cleaves peptide bonds at non-terminal amino acids like Glu, Met, Leu, and Tyr, enhancing protein functionality. Neutral protease selectively cleaves the bond between Leu and Phe at the C-terminal to expose amino acid residues and improve emulsifying activity through increasing the protein’s affinity for oil–water interfaces [23]. Cross-linking enzymes are able to strengthen the networks of protein molecules. Transglutaminase (TGase) is the most commonly used cross-linking enzyme, which can catalyze acyl transfer reactions between proteins or within proteins, resulting in covalent cross-linking between proteins [24]. The processing properties of proteins are significantly enhanced through modification with transglutaminase (TGase). For instance, it has been demonstrated that incorporating 0.8% TGase can increase the gel strength of pea protein isolate by approximately 4.2 times [25]. Additionally, the foam stability of whey protein isolate modified with TGase is extended by 100 min [26]. These data confirmed the significance of hydrolases and transglutaminase (TGase) in the improvement of the functional properties of proteins in food products. However, several studies also indicated that with the increase in the degree of hydrolysis, the bitter threshold decreased, and bitter taste increased. When the degree of hydrolysis exceeded 4%, the hydrolysates exhibited a relatively high intensity of bitterness [27]. The microbial TGase-mediated cross-linking process inherently possessed a debittering effect, further inhibiting the formation of bitter peptides [28]. However, enzymatic cross-linking may increase the particle size of the protein and have some negative effects on the properties of the protein. Consequently, researchers have begun combining enzymatic hydrolysis with enzymatic cross-linking to enhance the processing characteristics of protein molecules. Few studies have explored the use of enzymatic modification techniques to improve the emulsifying properties of YP.

Therefore, we aimed to combine enzymatic hydrolysis with enzymatic cross-linking modification to the emulsifying properties of YP primarily. We then utilized enzyme-modified YP (EYP) and two polysaccharides [including chitosan (CS, a cationic polysaccharide) and xanthan gum (XG, an anionic polysaccharide)] to fabricate complex Pickering emulsions to encapsulate MPs to improve its storage stability.

## 2. Materials and Methods

### 2.1. Materials

YP [protein content: 80% (*w*/*w*)] was provided by Angel Yeast Co., Ltd. (Hubei, China), while CS (degree of deacetylation ≥ 85%), XG, papain (1.05 × 10^5^ U/g), alkaline protease (2.0 × 10^5^ U/g), neutral protease (1.5 × 10^5^ U/g), and TG (10 U/g) were purchased from Jiahe Asahi Sunrise Co., Ltd. (Beijing, China). MPs (purity 90%) were obtained from Wanbang Biotechnology Co., Ltd. (Henan, China), and the sunflower seed oil was purchased from Cargill Co., Ltd. (Shanghai, China). Both MPs and sunflower seed oil were food grade, while other reagents were analytical grade. Deionized water was used throughout the experiment.

### 2.2. Optimization of Experimental Conditions for Enzymatic Modification of YP

Primarily, the effects of treating with TG and different hydrolases (including alkaline protease, papain, neutral protease) on the degree of hydrolysis, solubility, emulsifying activity index, and emulsifying stability index of YP were evaluated to determine the optimized hydrolase to be further applied in the enzymatic modification of YP. The preparation methods for the control group (Group 1) and the experimental group (Group 7) were as follows: yeast protein (YP) powder was dispersed in deionized water to prepare a 5% (*w*/*v*) YP solution, which was stirred at 1200 rpm at room temperature for 2 h; the pH was adjusted to 7.0 using 1 M HCl and 1 M NaOH; and precipitates were removed to obtain the control group solution. For the experimental group, the pH of the 5% (*w*/*v*) YP solution was adjusted to 7.0, 8.0, and 9.0, respectively, and papain, neutral protease, and basic protease were added to achieve an enzyme concentration of 4000 U/g; the solutions were enzymolized at 55 °C, 45 °C, and 40 °C, respectively. After 1 h of enzymolysis, the enzymes were deactivated by heating and boiling for 5 min, which has been shown to effectively denature the enzymes by reaching the temperature sufficient to disrupt their active sites [29]. The solutions were cooled in ice water at room temperature for 1 h, the pH was adjusted to 7.0, and precipitates were removed to obtain solutions of papain-modified yeast protein (P), alkaline protease-modified yeast protein (A), and neutral protease-modified yeast protein (N). Then, 10 U/g transglutaminase (TGase) was added to the control group and P, A, and N solutions, separately. The solutions were reacted at 40 °C for 1 h, followed by enzyme deactivation through heating and boiling for 5 min, and then cooled in ice water at room temperature for 1 h. The pH was adjusted to 7.0, and the final solutions were labeled as transglutaminase (TGase)-modified yeast protein (TG), papain and TGase complex-modified yeast protein (P-TG), alkaline protease and TGase complex-modified yeast protein (A-TG), and neutral protease and TGase co-modified yeast protein (N-TG). The specific detection methods for several indicators are as follows:

#### 2.2.1. Degree of Hydrolysis (DH) Assay

The degree of hydrolysis was measured using formaldehyde titration, and the specific operation steps were carried out by referring to previous methods [30]. The concentration of α-amino nitrogen was measured using formaldehyde titration, and the protein content was determined using an automatic nitrogen analyzer (KD-310, OPSIS, Stockholm, Sweden) according to GB/T 5009.235-2016. As commonly practiced in food science, the protein concentration was derived by applying a 6.25 multiplier to the total nitrogen measurement. The degree of hydrolysis was then determined by computing the ratio of α-amino nitrogen to total nitrogen, expressed as a percentage, as follows:

(1) $$DH\left(\%\right)=\frac{M_{0}}{M}\times 100\%$$

where *M_0_* is the mass of α-amino nitrogen, and *M* is the mass of total α-amino nitrogen.

#### 2.2.2. Solubility

Each protein sample was separately dissolved in deionized water to obtain a uni-form concentration of 1 mg/mL. After complete dissolution, the solutions were centrifuged at 4000 rpm for 15 min under refrigeration (4 °C) using the 3-30K type benchtop high-speed refrigerated centrifuge manufactured (Sigma, St. Louis, USA). The soluble protein content in the supernatant was tested with the BCA kit. The total protein content in the sample was measured with the automatic Kjeldahl nitrogen analyzer, and the solubility was calculated as the percentage of the protein content in the supernatant relative to the total protein content in the sample [8].

#### 2.2.3. Emulsifying Activity Index (EAI) and Emulsion Stability Index (ESI)

Based on the protocol of Yan [31], the emulsifying activity index and emulsion stability index of YP and EYP solutions were determined. A protein solution containing YP and EYP at a concentration of 0.1% (*w*/*v*) was prepared. Subsequently, 15 mL of this protein solution was combined with 5 mL of sunflower oil and homogenized for 1 min at a speed of 10,000 rpm. Specifically, 50 μL of the solution was removed from the bottom of the system and immediately added to 5 mL of sodium dodecyl sulfonate (SDS) aqueous solution (0.1%, *m*/*v*) at 0 min and 10 min, separately. An LAMBDA-750 ultraviolet spectrophotometer (PerkinElmer, Waltham, USA) was utilized to measure the absorbance values of different samples at 500 nm, while the SDS aqueous solution was used as blank. According to Equations (2) and (3), the emulsifying activity index and emulsion stability index of emulsions were calculated as follows:

(2) $$EAI\left(m^{2}/g\right)=\frac{2\times 2.303}{C\times \left(1-\theta \right)\times 10^{4}}\times A_{10}\times dilution\;factor$$

(3) $$ESI\left(\%\right)=\frac{A_{10}}{A_{0}}\times 100\%$$

where *A_0_* and *A_10_* were absorbance values of the sample at 0 and 10 min, respectively. In addition, *θ* was the percentage of sunflower seed oil in the sample, while *C* was the protein concentration (mg/mL).

Subsequently, to obtain the optimized experimental condition for the enzymatic hydrolysis with enzymatic cross-linking modification of YP, the effects of the additive amount of papain or transglutaminase as well as the reaction pH value, reaction temperature, reaction time of papain or transglutaminase with YP, and the emulsifying activity index and emulsion stability index of YP were investigated. YP solutions (5%, *w*/*v*) were prepared by dispersing YP powder in deionized water. Then, the mixtures were stirred for 2 h at 1200 rpm at room temperature. The pH value of different groups was adjusted to 6.0, 6.5, 7.0, 7.5, or 8.0, separately. Subsequently, 1000 U/g, 2000 U/g, 3000 U/g, 4000 U/g, or 5000 U/g of papain was added to different groups. The mixtures were kept at 45 °C, 50 °C, 55 °C, 60 °C, or 65 °C for 0.5 h, 1.0 h, 1.5 h, 2.0 h, or 2.5 h, respectively, after which the enzyme was inactivated by boiling for 5 min. When the mixtures were cooled to room temperature, the pH values of all samples was adjusted to 6.0, 6.5, 7.0, 7.5, or 8.0. Afterward, TG (5 U/g, 10 U/g, 15 U/g, 20 U/g, or 25 U/g) was added to different groups, and the mixtures were kept at 30 °C, 35 °C, 40 °C, 45 °C, or 50 °C for 0.5 h, 1.0 h, 1.5 h, 2.0 h, or 2.5 h, respectively. Ultimately, transglutaminase was inactivated by boiling for 5 min. After cooling down, all samples were centrifuged at 6000 rpm for 15 min. The supernatant of samples were collected, and the pH value was adjusted to neutral for further experiments.

### 2.3. Characterization of the Structural and Morphological Properties of EYP

The structural and morphological properties of EYP were characterized using ultraviolet–visible and fluorescence spectra, dynamic lighting scanning, Fourier transform infrared spectroscopy, gel permeation chromatography, electrophoresis, and scanning electron microscopy analysis.

#### 2.3.1. Ultraviolet–Visible and Fluorescence Spectra

The intrinsic fluorescence spectra and UV–vis spectra were determined based on the method reported by Ma et al. [20], with some modifications. The UV–vis spectra were measured using a LAMBDA-750 ultraviolet spectrophotometer (PerkinElmer, Waltham, USA). The protein sample concentration was adjusted to 3 mg/mL using deionized water, and the absorbance was recorded over a wavelength range of 230–380 nm. Background scanning was performed prior to the first sample measurement. All measurements were conducted in triplicate. The fluorescence spectra were determined using an FS5 fluorescence spectrophotometer (Applied Photophysics Ltd., Edinburgh, UK). The protein samples were diluted to 1 mg/mL with a 10 mM phosphate buffer (pH 7.0). The excitation wavelength was set at 265 nm, and the emission spectra were recorded over the range of 280–450 nm. The slit width was maintained at 5 nm for both excitation and emission. All measurements were performed in triplicate.

#### 2.3.2. Particle Size Distribution

The mean particle size of YP and EYP was determined using a Zetasizer Nano-ZS instrument (Microtrac MRB, Montgomeryville, PA, USA). Both native and modified yeast protein samples were prepared as 1 mg/mL aqueous solutions in deionized water. Measurements were performed at 25 °C after proper instrument equilibration.

#### 2.3.3. Fourier Transform Infrared Spectroscopy (FTIR)

The secondary structure of the protein was characterized using a Nicolet iS10 infrared spectrometer (Thermo Fisher Scientific, Waltham, MA, USA). Freeze-dried protein samples were mixed with dried KBr and thoroughly ground. The mixture was then pressed into pellets from the powder. The detection frequency range was 4000–500 cm⁻^1^, with a resolution of 4 cm⁻^1^. The infrared spectral curves were processed using Omnic 9.2 (Thermo Fisher Scientific Inc., Waltham, MA, USA) and Peakfit 4.12 (SPSS Inc., Chicago, IL, USA) for baseline correction, smoothing, Fourier deconvolution, and Gaussian peak fitting.

#### 2.3.4. Gel Permeation Chromatography

The relative molecular weight distribution of protein components was determined using a Shimadzu LC20 gel permeation chromatography. The samples were filtered through a 0.22 μm microporous filter membrane prior to analysis. The system was equipped with a RID-20A differential refractometer (Shimadzu Corporation, Kyoto, Japan) and a TSKgel GMPWXL aqueous gel column (300 mm × 7.8 mm, 13 μm). The mobile phase consisted of a 0.1 mol/L NaNO_3_ (Aladdin, Shanghai, China) solution containing 0.06% NaN_3_ (Aladdin, Shanghai, China), and the column temperature was maintained at 35 °C. The flow rate was set at 0.6 mL/min. The narrow molecular weight distribution of pullulan polysaccharide was used as the calibration standard, and the relative correction method was employed for data analysis.

#### 2.3.5. Sodium Dodecyl Sulfate-Polyacrylamide Gel Electrophoresis (SDS-PAGE)

To characterize the protein degradation in yeast protein samples under reducing conditions, the Ma method was employed [20]. The samples, containing different types of proteins, were analyzed using SDS-PAGE (Bio-Rad Laboratories, Hercules, CA, USA) with 12% and 5% resolving gels for enrichment and separation of protein peptides, respectively. For the 5% protein sample supernatant, an equal volume of 5% (*v*/*v*) β-mercaptoethanol (Sigma-Aldrich, St. Louis, MO, USA) loading buffer was added to achieve the final concentration. The mixture was then heated in boiling water for 10 min. After cooling to room temperature, 20 µL of the prepared sample was loaded onto the gel. Electrophoresis was performed at a constant voltage of 90 V to separate the standard protein bands, followed by a regulated voltage of 120 V until the tracking dye reached the bottom of the gel. The molecular weight of the proteins was compared to a low molecular weight standard (11–245 kDa) protein marker. After electrophoresis, the gel was removed and stained in a solution containing 0.25% (*w*/*v*) Coomassie Brilliant Blue R-250 for 40 min. The gel was then destained in a solution containing 25% methanol and 7% acetic acid until the protein bands were clearly visible. Finally, the gel was imaged using a gel documentation system.

#### 2.3.6. Scanning Electron Microscopy

The samples were individually freeze-dried. The freeze-dried samples were then mounted onto the scanning electron microscopy observation platform and sputtered with ions. The samples were placed on the sample holder approximately 10–15 cm away from the evaporation source and rotated during the sputtering process. Gold sputtering was performed at 10 kV for 60 s to ensure an even coating. After the coating was completed, the samples were examined under the microscope. Subsequently, the samples were coated with gold using a sputter coater. The morphology of the samples was examined using scanning electron microscopy (Thermo Fisher Scientific, Hillsboro, OR, USA) at magnifications of 3000× and 6000×.

### 2.4. Optimization of Experimental Conditions for the Preparation of YP– and EYP–Polysaccharide Complex Pickering Emulsions

Briefly, 2.0 g of CS or XG was dissolved in 100 mL of deionized water and stirred at room temperature for 4 h. The YP– and EYP–polysaccharide complex Pickering emulsions were prepared according to the method of Zeng et al. (2024), with some modifications [32]. Briefly, YP or EYP solutions were mixed with CS or XG solutions at 1:1, separately, and then the mixtures were heated at 90 °C for 8 min. When YP–CS and EYP–CS or YP–XG and EYP–XG mixtures were cooled down, the pH values of YP–CS and EYP–CS or YP–XG and EYP–XG mixtures were adjusted to 2.5, 3.5, 4.5, 5.5, or 6.5 and 3.5, 5.5, 6.5, or 7.5, respectively, using HCl (1%, *v*/*v*) and NaOH (1%, *w*/*v*) solutions. Subsequently, sunflower seed oil was added to the mixtures of YP/EYP–CS and YP/EYP–XG to maintain the volume ratio of the oil phase of the emulsions at 40%. Ultimately, the mixtures were stirred at 12,000 rpm for 4 min to obtain the final Pickering emulsions. The final volume was considered unchanged, as the system was closed and evaporation during the short period of high-speed stirring could be negligible. To investigate the effects of different mass ratios on the formation of YP– and EYP–polysaccharide complex Pickering emulsions, YP or EYP solutions were mixed with CS or XG solutions at different mass ratios (5:1, 3:1, 1:1, 1:3, 1:5). While the pH values and the volume ratio of oil phase in the emulsions of YP–CS and EYP–CS or YP–XG and EYP–XG were kept at 3.5 or 5.5 and 40%, respectively. Moreover, the mass ratio of protein and polysaccharide as well as the pH value of YP–CS and EYP–CS or YP–XG and EYP–XG mixtures were maintained at 1:1 and 5.5 or 3.5 when analyzing the effects of volume ratio of sunflower seed oil in the emulsions (20%, 30%, 40%, 50%, 60%) on the formation of complex Pickering emulsions. To obtain the optimized experimental condition for preparation of YP– and EYP–polysaccharide complex Pickering emulsions, the particle size, zeta potential, emulsifying activity index, emulsion stability index, and creaming index of emulsions were selected as the indicators. Here, pH 5.5 and 3.5 were selected based on the good stability of protein–polysaccharide complexes under acidic conditions. These values referred to the initial pH before emulsification, which slightly increased after oil addition due to buffering.

The particle size and zeta potential of emulsions were measured using the method described by [33]. Briefly, the emulsions were diluted 160 times with deionized water and then measured using a nanoparticle particle size analyzer (Malvern, Nano-ZS90, Worcestershire, UK). Based on the protocol of Yan [31], the emulsifying activity index and emulsion stability index of YP/EYP–CS and YP/EYP–XG complex Pickering emulsions were determined. Specifically, 10 μL of emulsion was removed from the bottom of the system and immediately added to 5 mL of sodium dodecyl sulfonate (SDS) aqueous solution (0.1%, *m*/*v*) at 0 min and 10 min, separately. An Ultrospec 2100 pro spectrophotometer (General Electric Company, London, UK) was utilized to measure the absorbance values of different samples at 500 nm, while the SDS aqueous solution was used as blank. According to Equations (1) and (2), the emulsifying activity index and emulsion stability index of emulsions could be calculated.

Furthermore, the creaming index (CI) of emulsion was determined based on the method of [33], with minor modifications. Two layers (creaming layer and water) were measured after placing the samples in a water bath of 85 °C for 5 h. Then, the CI of the samples was measured using Equation (4):

(4) $$CI\left(\%\right)=\frac{H_{c}}{H_{t}}\times 100\%$$

where *H_c_* and *H_t_* are the heights of the creaming layer and whole system, respectively.

### 2.5. Optimization of Experimental Conditions for the Preparation of MP-Loaded YP– and EYP–Polysaccharide Complex Pickering Emulsions

YP or EYP solutions (2% *w*/*v*) were mixed with CS or XG solutions (2% *w*/*v*) at a mass ratio of 1:1, separately. Then, the mixtures were heated at 90 °C for 8 min. When YP–CS and EYP–CS or YP–XG and EYP–XG mixtures were cooled down, their pH values were adjusted to 5.5 or 3.5 with HCl (1%, *v*/*v*) and NaOH (1%, *w*/*v*) solutions, respectively. Subsequently, 100, 150, 200, 250, or 300 µL of MP (15%, *w*/*v*) aqueous solution was added to 15 mL of YP–CS and EYP–CS or YP–XG and EYP–XG mixture, and then 10 mL (40%) or 15 mL (50%) of sunflower oil was added to these samples. Ultimately, the mixtures were stirred at 12,000 rpm for 4 min for further analysis.

The optimized experimental conditions for the preparation of MP-loaded complex Pickering emulsions were determined by the encapsulation rate (EE) of MPs in emulsions. Based on the method of [34], 4 mL of emulsion was diluted to 12 mL using deionized water and then freeze-centrifuged at 10 °C for 30 min at 4000× *g* to separate MPs in the emulsion. The lower layer was filtered through a 0.22 μm PES microporous filter membrane. Ultimately, the absorbance of the sample was measured using a UV–vis spectrophotometer at 423 nm. The encapsulation rate of the emulsion was calculated according to Equation (5) as follows:

(5) $$EE\left(\%\right)=\frac{C_{i}-C_{0}}{C_{i}}\times 100\%$$

where *C_i_* was the additive concentration of MP, and *C_0_* represented the concentration of MPs in the outer aqueous phase after centrifugation.

### 2.6. Characterization of Different MP-Loaded Complex Pickering Emulsions

The physicochemical and structural properties of MP-loaded complex Pickering emulsions were characterized using rheological, Raman spectroscopy, and confocal laser scanning microscope (CLSM) analysis. Specifically, the rheological properties of emulsions were determined with an Antonpa rheometer using a PP50 parallel plate geometry measurement system (Graz, Austria). The parameters were set as follows: gap size: 1 mm, measuring temperature: 5 °C. The viscosity of samples was measured at a shear rate of 0.1–100 s^−1^, while the storage modulus (G’) and loss modulus (G”) were obtained at an angular frequency of 0.1 ~ 10 rad/s under 1% stress.

Regarding Raman spectroscopy analysis, a benchtop MacroRAM model Raman spectrometer (Horiba, Kyoto, Japan) was used as the excitation source with a near-infrared laser operating at 785 nm and a laser power of 40%, while the scanning range and data acquisition time were 400–3500 cm^−1^ and 60 s, respectively. The raw spectra were corrected using the LabSpec 6 program, and the Peakfit 4.12 program was utilized to calculate the secondary structure content in different samples.

The interfacial structures of emulsions were visualized using confocal laser scanning microscopy (TCSSP8, Leica, Wetzlar, Germany) according to the method of previous studies, with some modifications [19,32]. Fluorescein isothiocyanate (FITC) was used to stain aqueous phase in samples, while YP/EYP and the oil phase were stained with Nile blue or Nile red, respectively. Specifically, 10 μL of dye (1 mg/mL) was added to 400 μL of emulsions to stain different components and then transferred to the dark for 10 min. Afterwards, 5 μL of the stained sample was carefully placed on a microscope slide and covered with a coverslip, ensuring no trapped air gaps or bubbles between the mixture and the coverslip. Before observation, the mixtures were equilibrated for 2 min. Images of Nile red (oil phase), Nile blue (protein), and FITC (aqueous phase) were captured at excitation wavelengths of 488 nm, 633 nm, and 490 nm, respectively.

### 2.7. Stability of Different MP-Loaded Complex Pickering Emulsions During Storage at Room Temperature

The stability of different MP-loaded complex Pickering emulsions during storage was evaluated using color analysis and by determining the retention rate of MPs in emulsions at different conditions. On the one hand, the color changes of different samples during storage was measured using the SC-10 Chroma Meter (3NH Technology, Guangzhou, China) based on the method of Mohammed [35], with minor modifications. Briefly, 3 mL of samples or MP solution were transferred to a clear petri dish. On the 0th, 2nd, 4th, 6th, 8th, and 10th days of storage at room temperature, L* (lightness), a* (redness/greenness), and b* (yellowness/blueness) values were recorded to estimate the change in color ΔE based on Equation (6):

(6) $$\Delta E=\sqrt{{\left(L^{*}-{L_{0}}^{*}\right)}^{2}+{{\left(a^{*}-{a_{0}}^{*}\right)}^{2}+(b^{*}-{b_{0}}^{*})}^{2}}$$

where L*, a*, and b* are the values of samples stored for different times, while L_0_*, a_0_*, and b_0_* represent the values of samples at day 0.

On the other hand, the effects of storage and heating time on the stability of MPs in the emulsions were investigated according to the method described by [36]. To be specific, the effects of storage time (0–8 d) on the retention rate of MPs in emulsions at 4 °C in the dark were studied. Moreover, the retention rate of MPs in emulsions was measured after being heated in a water bath at 80 °C for 180 min. During heating, the retention rate of MPs in the sample was determined every 30 min during heat treatment. The emulsion was diluted 20 times using deionized water, while MPs in the emulsion were separated by centrifuging at 6000× *g* for 30 min. Ultimately, the MP concentration in the supernatant was detected using UV–vis spectrophotometry. The retention rate of MPs in emulsion was calculated using Equation (7):

(7) $$\mathrm{Retention}\;\mathrm{rate}\;\left(\%\right)=\frac{A_{1}}{A_{0}}\times 100\%$$

where *A_1_* represented the absorbance of MPs after storage, and *A_0_* indicated the initial absorbance of MP.

### 2.8. Statistical Analysis

All the experiments were performed in triplicate, and the results were expressed as the means ± standard deviation (SD) using SPSS 17.0 (USA) for Windows. The statistical significance of data was determined using a one-way analysis of variance (ANOVA) followed by the comparison of Duncan’s multiple range test (DMRT), and *p* values <0.05 were regarded as significant.

## 3. Results and Discussion

### 3.1. Effects of Different Enzymatic Modification Treatments on the DH, Solubility, EAI, and ESI of YP

During enzymatic modification, the degree of hydrolysis (DH) is a critical parameter for controlling product quality. Real-time monitoring of the degree of hydrolysis can help avoid both over-hydrolysis and under-hydrolysis, ensuring the stability and consistency of the final product. As illustrated in Figure S1A, the degree of hydrolysis of proteins modified with papain (papain-modified yeast protein and papain and TGase complex-modified yeast protein) was found to be 0.77% and 0.92%, respectively. These values were significantly lower than those obtained with alkaline protease and neutral protease. This finding suggests that papain may be a more suitable enzyme for achieving the desired degree of hydrolysis while minimizing the risk of generating bitter peptides.

The solubility of protein is a fundamental aspect of its functional properties. Enzymatic modification, through partial hydrolysis, exposes more polar groups and hydrophobic residues in the protein structure. This structural alteration enhances the protein’s interaction with water molecules, thereby significantly influencing its solubility. As shown in Figure S1B, the solubility of sample alkaline protease and TGase complex-modified yeast protein was 7.2%, which is 1.81 times higher than that of papain and TGase complex-modified yeast protein. Similarly, the solubility of papain and TGase complex-modified yeast protein was also 7.2%, representing a 1.10-fold increase compared to alkaline protease and TGase complex-modified yeast protein. These results indicate that enzymatic modification effectively improved the solubility of the proteins, with Alkaline protease and TGase complex-modified yeast protein demonstrating the most pronounced enhancement.

In addition, studies show that the solubility of hydrolyzed chickpea protein increases while the interfacial tension decreases. At a lower degree of hydrolysis, the emulsification ability can be increased [37]. Emulsifying ability and emulsifying stability are two important indexes to measure the emulsifying properties of protein, which reflect the ability of protein to form and maintain emulsion in an oil–water system. Moderate hydrolysis can increase the surface hydrophobicity and charge density of the protein, thereby improving its adsorption capacity and emulsification stability at the oil–water interface. For example, Wang found that rice bran protein with different enzymatic hydrolysis degrees showed significant differences in emulsification activity and stability, and the moderately hydrolyzed protein had better emulsification performance [7]. As can be seen in Figure S1C, the emulsification activity index values of the papain-modified protein (papain-modified yeast protein and papain and TGase complex-modified yeast protein) were 74.74 m^2^/g and 73.33 m^2^/g, respectively, which was significantly higher than that of other sample groups and 1.68 and 1.65 times that of alkaline protease and TGase complex-modified yeast protein, respectively. In addition, it can be seen that the emulsion stability index of the papain and TGase complex-modified yeast protein of the sample is second only to that of the control group, and there is no significant difference between the two groups.

In summary, limited hydrolysis significantly enhances the emulsification and solubility properties of proteins. Among the samples tested, papain-modified yeast protein and papain and TGase complex-modified yeast protein exhibited superior emulsification abilities. Notably, the solubility and emulsifying stability index of papain and TGase complex-modified yeast protein were higher than those of papain-modified yeast protein. These results suggest that the combined treatment with papain and transglutaminase enzyme is an effective approach for modifying yeast protein. This method not only improves the emulsification properties but also enhances the solubility of the protein, making it a suitable choice for applications requiring high functional performance.

### 3.2. Optimization of Experimental Conditions for Enzymatic Hydrolysis with Enzymatic Cross-Linking Modification of YP

The optimization of yeast protein modification conditions was conducted using the emulsifying activity index and emulsifying stability index as evaluation criteria. As illustrated in Figure S2, when papain was added at a concentration of 3000 U/g, the emulsifying activity index increased from 47.53 m^2^/g to 83.03 m^2^/g, and the emulsifying stability index rose from 60.73% to 65.26%. Both of these values were significantly higher than those of the unmodified yeast protein (YP). Under pH 7 conditions, the emulsifying activity index and emulsifying stability index further increased to 77.38 m^2^/g and 63.21%, respectively. Similarly, at 65 °C, the emulsifying activity index and emulsifying stability index reached 81.76 m^2^/g and 67.85%, respectively. After 1.5 h of enzymolysis, the emulsifying activity index and emulsifying stability index were 80.28 m^2^/g and 63.46%, respectively. All of these values represent substantial improvements over YP.

As shown in Figure S3, the addition of 10 U/g transglutaminase enzyme also led to notable enhancements. Compared with YP, the emulsifying activity index increased from 56.13 m^2^/g to 87.94 m^2^/g, and the emulsifying stability index rose from 50.65% to 59.03%. Under pH 8 conditions, the emulsifying activity index and emulsion stability index reached 83.42 m^2^/g and 68.22%, respectively. At 40 °C, the emulsifying activity index and emulsifying stability index were 77.03 m^2^/g and 53.78%, respectively. After 1 h of cross-linking, the emulsifying activity index and emulsifying stability index reached 94.68 m^2^/g and 59.27%, respectively.

In summary, the optimal conditions for enzymatic modification of yeast protein were determined to be the following: papain at 3000 U/g, pH 7, 65 °C, and 1.5 h of enzymolysis; followed by TG enzyme at 10 U/g, pH 8, and 40 °C for 1 h of cross-linking. Under these conditions, the modified yeast protein (EYP) exhibited an emulsifying activity index that was 1.83 times higher and an emulsifying stability index that was 1.31 times higher than those of the unmodified yeast protein (YP).

### 3.3. Structural and Morphological Properties of EYP

#### 3.3.1. Ultraviolet–Visible and Fluorescence Spectra of YP and EYP Protein Solutions

UV–vis and fluorescence spectroscopy are sensitive to the presence of aromatic amino acids in proteins, and spectral changes reflect conformational alterations in protein structure. As depicted in Figure S4, the yeast protein exhibited a UV absorption peak near 260 nm, consistent with previous findings [20]. This absorption is attributed to the aromatic heterocycles of tyrosine, tryptophan, and phenylalanine within the protein structure, which absorb UV light in this region and undergo π→π* transitions. Notably, the intensity of the absorption peak increased significantly following the modification of yeast protein (YP). This enhancement suggests that the conformation of YP has changed, thereby affecting the microenvironment surrounding these aromatic amino acid residues [38].

Endogenous fluorescence spectroscopy is a powerful tool for monitoring the tertiary structure of proteins, primarily by detecting the intrinsic fluorescence emitted by tryptophan residues. This technique can sensitively reflect changes in the polarity of the microenvironment surrounding proteins [25]. As shown in Figure S5, the yeast protein exhibited a maximum fluorescence intensity at 340 nm both before and after modification. However, a significant increase in fluorescence intensity was observed post-modification. This enhancement indicates that the modification process facilitated the exposure of tryptophan residues from a hydrophobic environment to a more aqueous environment. This finding is consistent with the results obtained from the UV spectrum analysis, further supporting the structural changes induced by the modification.

#### 3.3.2. Size Distribution of YP and EYP Protein Solutions

As depicted in Figure S6, the particle size distributions of YP and EYP exhibit marked differences. YP displays a larger average particle size with a more dispersed distribution, characterized by three distinct peaks. In contrast, the modified EYP sample features two peaks and a more compact particle size distribution. The smaller particle size of EYP is advantageous in reducing particle aggregation under thermal stress, thereby enhancing the thermal stability of yeast protein particles [39].

#### 3.3.3. Fourier Transform Infrared Spectroscopy of YP and EYP Protein Solutions

Infrared spectroscopy is very sensitive to conformational changes of proteins, and the amide I band is very sensitive to changes in hydrogen bond patterns. This property allows infrared spectroscopy to be used to monitor conformational changes in proteins under different conditions, such as structural changes during heat treatment, chemical modification, or aggregation [40]. Figure S7 shows the infrared spectra of YP and EYP. YP and EYP show a wide protein N-H stretching vibration peak at 3278.28 cm^−1^ and a low protein single bond stretching vibration peak at 1044.39 cm^−1^ and 1012.17 cm^−1^. The C=O, N-H, and C-N stretching vibration peaks appear in the amide I band (1700–1600 cm^−1^), amide II band (1600–1500 cm^−1^), and amide III band (1300–1200 cm^−1^), respectively. To gain deeper insights into the structural changes, we conducted a detailed analysis of the peak values of the amide I band. Further analysis of the peak value of the amide I band showed that enzymatic modification reduced the β-sheet and random coil structure content in yeast protein, but increased the α-helix and β-turn structure content, as shown in Table S1. These findings have been independently validated by other research groups, providing further evidence that the enhancement in solubility and emulsification properties can be attributed to structural modification [41].

#### 3.3.4. Gel Permeation Chromatography of YP and EYP Protein Solutions

As a robust analytical technique, gel permeation chromatography is extensively employed to determine the molecular weight and distribution of proteins, owing to its high efficiency and accuracy. In this study (Table S2), gel permeation chromatography analysis revealed distinct differences in the molecular weight distribution of peptides between YP and EYP. Specifically, the molecular weight distribution of YP peptides exhibited a relatively broad profile with four distinct peaks, whereas EYP displayed three peaks with an increased abundance of both high- and low-molecular-weight peptides. These findings suggest that the modification process significantly altered the molecular weight distribution of yeast protein, likely through the cleavage or reformation of peptide bonds. This redistribution may have important implications for the functional properties of the modified yeast protein, which warrants further investigation.

#### 3.3.5. Sodium Dodecyl Sulfate-Polyacrylamide Gel Electrophoresis

To elucidate the impact of enzymatic modification on protein degradation in the samples, sodium dodecyl sulfate-polyacrylamide gel electrophoresis analysis was performed under reducing conditions. As depicted in Figure S8, the molecular weight distribution of YP is primarily concentrated within the ranges of 11–25 kDa, 35–48 kDa, and 100–245 kDa, with several minor bands also observed. In contrast, the high-molecular-weight protein band at 245 kDa in EYP was notably diminished compared to YP. This reduction is likely attributed to the cleavage of peptide bonds in high-molecular-weight proteins during enzymatic hydrolysis, resulting in the formation of smaller peptides and protein fragments. Lower-molecular-weight protein fragments generally exhibit higher surface activity and can more efficiently adsorb at the oil–water interface, thereby enhancing emulsification stability. This phenomenon was well-documented in the literature, where enzymatic hydrolysis or other treatments of plant proteins were shown to reduce their molecular weights and significantly improve their emulsifying properties [42]. These findings provide a plausible explanation for the enhanced emulsification stability observed in our experiments.

#### 3.3.6. Scanning Electron Microscopy of YP and EYP Protein Solutions

The microstructural characteristics of lyophilized proteins were examined. As depicted in Figure S9, the YP supernatant exhibited a sheet-like structure with varying sizes, shapes, and rough surfaces, whereas the EYP supernatant particles displayed a uniform network structure characterized by larger pore sizes. These observations suggest that papain hydrolysis of yeast protein generates more soluble peptides, thereby exposing additional lysine residues. The increased availability of lysine residues facilitates cross-linking by transglutaminase enzyme, promoting the formation of ε-(γ-glutamyl) lysine covalent bonds. This enzymatic cross-linking ultimately leads to significant alterations in the microstructure of the proteins.

### 3.4. Optimization of the Preparing Process of YP– and EYP –Polysaccharide Complex Pickering Emulsions

To obtain the optimal experimental conditions for the preparation of YP–CS and EYP–CS as well as YP–XG and EYP–XG Pickering emulsions, the effects of pH, protein/polysaccharide mass ratio, and the additive volume of oil on the particle size distribution, zeta potential, emulsifying activity index, emulsion stability index, and creaming index of emulsions were investigated. Particle size and zeta potential are important parameters that indicate the stability of emulsions. Generally, the particle size of samples suggested the strength of mutual repulsion or attraction between particles, while the emulsion with a smaller particle size shows a higher absolute value of zeta potential and lower possibility of aggregation or coagulation between particles [43]. When the absolute value of the zeta potential of the emulsion is higher than 30 mV, it has good stability [44]. The emulsifying activity index represents the relative surface coverage of protein molecules on oil droplets in an emulsion system, and the emulsion stability index is an effective parameter to evaluate the relative stability of biopolymer-stabilized emulsions after storage for a period of time [12]. Furthermore, the creaming index reflects the stability of emulsions by measuring the ratio of the height of the upper emulsion layer and the overall height of the emulsion.

The interactions of proteins with polysaccharides mainly originates from the electrostatic interactions between oppositely charged polymers. The pH value of the system is one essential factor that influences the electrostatic interactions between proteins and polysaccharides. The pH value can alter the charge state, molecular conformation, and surface properties of biopolymers and further affect the stability and properties of emulsions. As shown in Figure S10A,D of the Supplementary Materials, the average particle sizes of YP–CS and EYP–CS emulsions fabricated at a pH value of 4.5 were larger than those fabricated at other pH values, while YP–XG and EYP–XG emulsions had the largest average particle sizes when prepared at pH values of 4.5 and 6.5, respectively. It could be observed that the average particle size of EYP-based emulsions prepared at different pH values were significantly smaller than that of YP-based emulsions given that enzyme modification greatly decreased the average particle size of YP [45]. For instance, the average particle size of EYP–CS emulsions fabricated at a pH value of 4.5 was reduced to 66.6% of the original YP–CS particle size. In addition, the average particle size of CS-based emulsions was smaller compared to that of XG-based emulsion. This phenomenon may be attributed to the stronger electrostatic interactions between CS and yeast proteins compared to XG, which facilitates a more uniform distribution of CS on the emulsion droplet surfaces [33]. In accordance with the results of particle size analysis, the absolute values of zeta potential of the protein–polysaccharide complex Pickering emulsions fabricated at a pH value of 4.5 were the highest among all groups (Figure S10B,E of the Supplementary Materials). The absolute value of zeta potential of EYP–CS Pickering emulsions were relatively higher than that of YP–CS emulsions. Notably, the zeta potential of EYP–CS Pickering emulsions fabricated at a pH value of 4.5 was 1.02 times that of YP–CS emulsions. As evidenced by Figure S10C,F, CS-based emulsions showed peak emulsifying activity at pH 5.5, whereas XG-based emulsions attained maximum emulsifying activity at pH 3.5. It was worth mentioning that the emulsifying activity index of EYP–XG emulsions fabricated at a pH value of 3.5 was 1.91-fold higher compared to that of YP–XG emulsions. Due to the enzymatic modification, the emulsifying activity index of EYP-based emulsions was relatively higher as compared with that of YP-based emulsions. Regarding emulsion stability index analysis, the emulsion stability index of YP- CS and YP–XG emulsions was the highest when prepared at a pH value of 6.5 and 7.5, respectively, while the emulsion stability index of EYP–CS and EYP–XG emulsions reached peak values at pH values of 5.5 and 4.5, respectively. As shown in Table S3 of the Supplementary Materials, the creaming index of CS-based emulsions fabricated at a pH value of 2.5 was the lowest, while XG-based emulsions prepared at a pH value of 6.5 exhibited significantly lower creaming index values than other groups, suggesting that the stability of CS-based and XG-based emulsions was the lowest when prepared at pH values of 2.5 and 6.5, respectively. Notably, the creaming index of EYP–CS emulsions prepared at pH 2.5 was only 57.14% of that observed for emulsions prepared at pH 6.5. Combining the findings of particle size, zeta potential, emulsifying activity index, emulsion stability index, and creaming index, optimal pH values for the preparation of the YP–CS or EYP–CS as well as YP–XG or EYP–XG emulsions were 5.5 and 3.5 for subsequent studies.

Figure S11 of the Supplementary Materials shows the effects of protein/polysaccharide mass ratio on different physicochemical properties of complex Pickering emulsions. The average particle sizes of both CS-based and XG-based emulsions were the smallest when the protein and polysaccharide mass ratio was 1:1 and 3:1, respectively, indicating that the intermolecular force between biopolymers was relatively balanced at this ratio (Figure S11A,D of the Supplementary Materials). At a protein/polysaccharide mass ratio of 1:1, the average particle size of EYP–CS emulsions was 75.23% that of EYP–XG emulsions. In terms of the zeta potential, with the decrease in the protein/polysaccharide mass ratio, the zeta potential of emulsions was relatively stable. It could be observed in Figure S11B,E of the Supplementary Materials that the absolute value of zeta potential of CS-based emulsions was higher compared with that of XG-based emulsions, while EYP-based emulsions had a relatively higher absolute zeta potential value than YP-based emulsions. The phenomenon suggested that the enzymatic modification of YP might increase the stability of complex Pickering emulsions. With the increase in the polysaccharide/protein mass ratio, the emulsifying activity index of emulsions increased and then decreased, while the change in emulsion stability index showed an opposite tendency (Figure S11C,F of the Supplementary Materials). The emulsifying activity index of EYP–CS emulsions was 1.39 times higher than that of YP–CS emulsions when the protein/polysaccharide ratio was 1:1. Moreover, as shown in Table S3 of the Supplementary Materials, YP–CS and/EYP–CS emulsions were unstable when the protein/polysaccharide mass ratio was lower than 1. However, the YP–XG and EYP–XG emulsions were still thermally stable at a high concentration of polysaccharide, possibly because the high viscosity of XG prevented the movement between droplets. At a protein/polysaccharide mass ratio of 1:1, the creaming index of EYP–CS emulsions was only 60.38% that of EYP–XG emulsions. Overall, the optimal protein/polysaccharide mass ratio was selected as 1:1 for the preparation of both CS- and XG-based emulsions in the following studies.

Further, the effects of the additive volume of oil on different properties of emulsions were investigated as well. Figure S12A,D of the Supplementary Materials shows that the average particle size of emulsions was the lowest when the oil additive volume was 20% (*v*/*v*). To be specific, the mean particle diameter of EYP–XG emulsions containing 60% (*v*/*v*) oil phase was 1.96 times greater than those with 20% (*v*/*v*) oil content. As the volume fraction of the internal phase increased, the droplets were more tightly arranged and interconnected, forming a dense reticulated oil droplet-filling system, the size of which became progressively smaller [46]. At a high oil additive volume, the complex particles were unable to cover the droplet surface uniformly, ultimately leading to droplet flocculation during homogenization [47,48]. The zeta potential of emulsions remained relatively stable with the increase in the oil additive volume, while the absolute zeta potential values of EYP-based emulsions were significantly higher compared with that of YP-based emulsions (Figure S12B,E of the Supplementary Materials). Moreover, the absolute value of zeta potential of CS-based emulsions were higher as compared to that of XG-based emulsions. When the oil additive volume was 40% (*v*/*v*), the zeta potential of EYP–CS emulsions was 4.27 mV higher than that of YP–CS emulsions. Regarding the emulsifying activity index of emulsions, the emulsifying activity index values of EYP–CS, YP–CS, and EYP–XG emulsions reached the peak when the additive volume of oil was 50% (*v*/*v*), whilst the emulsifying activity index value of YP–XG emulsions gradually increased with the increase in the additive volume of oil (Figure S12C,F of the Supplementary Materials). The emulsifying activity index of EYP–CS emulsions was 11.68 m^2^/g lower than that of EYP–XG emulsions. Notably, the change in the emulsion stability index of XG-based emulsions with the increase of additive oil volume was similar to that noted for their emulsion stability index. However, the emulsion stability index of YP–CS emulsions showed the opposite tendency of variation. At an additive volume of oil of 50% (*v*/*v*), the emulsion stability index value of EYP–CS emulsions was 92.09%that of EYP–XG emulsions. As shown in Table S2 of the Supplementary Materials, all emulsions exhibited a high stability when the additive volume of oil was higher than or equal to 50%, possibly on account of the enhancement of the viscosity of emulsions, which restricted the flow of oil droplets and prevented their aggregation [49]. In summary, the optimal additive volume of oil for fabrication of CS-based and XG-based emulsions was 40% (*v*/*v*) and 50% (*v*/*v*), respectively, in subsequent studies.

### 3.5. Effects of MP Concentration on the Encapsulation Efficiency (EE) for MPs in YP– and EYP–Polysaccharide Complex Pickering Emulsions

To determine the optimal experimental condition to prepare the MP-loaded emulsions, the effects of MP concentration on the encapsulation efficiency for MPs in emulsions were studied in our research. Table 1 shows that the encapsulation efficiency for MPs of CS-based emulsions was significantly higher than that of XG-based emulsions. With the increase in MP concentration, the encapsulation efficiency for MPs in emulsions increased and then decreased. Moreover, the encapsulation efficiency for MPs in EYP-based emulsions reached the peak when the MP concentration was 0.2% (*w*/*v*). At a MP concentration of 0.2% (*w*/*v*), the encapsulation efficiency for MPs in EYP–CS emulsions was 3.09% higher compared with that in YP–CS emulsions. On the one hand, the electrostatic interactions of proteins with cationic CS were much stronger than XG, which might help to form a more stable emulsion interface and thus improve the MP encapsulation efficiency. On the other hand, XG was more viscous than CS, which made it more difficult to form a homogeneous emulsion and led to a lower encapsulation efficiency for MPs. Thus, in the following studies the optimal MP concentration for the preparation of MP-loaded emulsions was 0.2% (*w*/*v*).

### 3.6. Rheological Properties of MP-Loaded Complex Pickering Emulsions

**Figure 1:**
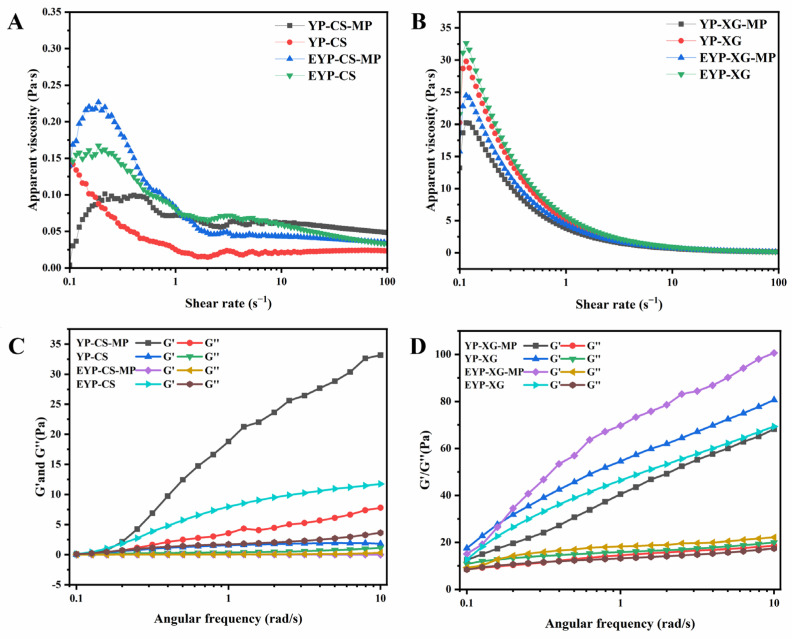
Static and dynamic rheological properties of CS-based (**A**,**C**) and XG-based (**B**,**D**) complex Pickering emulsions.

Rheological properties are regarded as important indicators for evaluating the appearance and stability of emulsions [50]. The static and dynamic rheological properties of different emulsions are shown in Figure 1. The apparent viscosity of XG-based emulsions was significantly higher than that of CS-based emulsions, while the viscosity of emulsions decreased continuously with the increase in shear rate, manifesting a shear thinning phenomenon (Figure 1A,B). The molecular structure of XG contained a large number of side chains, which could interact with each other to form a network structure. In contrast, the molecular structure of chitosan was relatively simple, leading to a low initial viscosity. With the enhancement of shear stress, the rate of intermolecular destruction was higher than the rate of re-formation, and the emulsions showed the phenomenon of droplet deformation and network breakage, which resulted in a low flow resistance and apparent viscosity. In addition, the apparent viscosity of EYP-based emulsions were remarkably higher compared with YP-based emulsions, whilst MP-loaded emulsions had a relatively higher viscosity than control based on CS. MP-loaded emulsions had a relatively lower viscosity than control based on XG. According to Shi et al. (2024), high emulsion viscosity was correlated with hindered particle motion and high stability [51]. Hence, the enzymatic modification and MP encapsulation treatment might increase the stability of CS-based and XG-based emulsions.

The dynamic moduli (G’ and G’’) could be used to characterize the degree of cross-linking in the network of emulsion systems, where G’ or G’’ represent elasticity and viscosity, respectively. Figure 1C,D show that the values of G’ and G’’ of all samples except MP-loaded EYP–CS emulsions gradually increased with the increase in frequency (0.1–10 rad/s), exhibiting elastic-dominated solid-like behavior characteristics. By contrast, the G’ and G’’ curves of MP-loaded EYP emulsions showed a significant crossover with increasing frequency rate, indicating that the emulsion changed from a gel to a liquid-like state [52]. In addition, the G’ and G’’ values of XG-based emulsions were significantly higher in comparison with those of CS-based emulsions, which is attributed to their stronger viscoelastic properties. The loss factor tan *δ* is the ratio of G’’ to G’. When tan *δ* was greater than 1, the viscosity of sample was greater than elasticity. As shown in Table 2, at an angular frequency of 1 rad/s, tan *δ* values for all samples were less than 1, suggesting their higher elasticity. The tan *δ* values of emulsions increased after the encapsulation of MPs, and it seemed that addition of MPs improved the viscosity of emulsions [53].

### 3.7. Raman Spectrum Analysis of MP-Loaded Complex Pickering Emulsions

**Figure 2:**
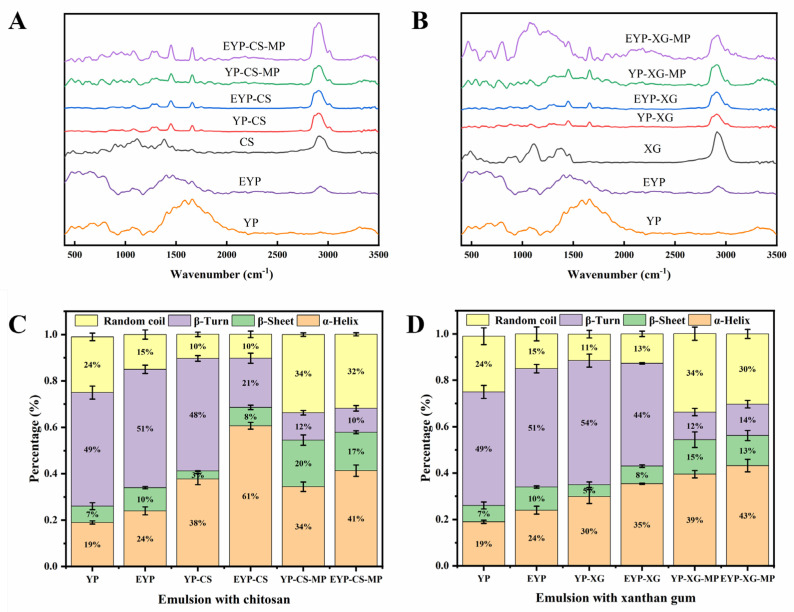
The Raman spectrum and secondary structure of CS-based (**A**,**C**) and XG-based (**B**,**D**) complex Pickering emulsions.

The changes in protein secondary structures in samples were investigated using Raman spectrum analysis. Figure 2A,B presents the Raman spectra of CS-based and XG-based emulsions, respectively. The amide I band (1700–1600 cm^−1^) in the spectra was used to extract the information on the secondary structures of YP and EYP, which represent the C-O stretching vibration of the amide moiety as well as the in-plane bending of N-H and the stretching of the C-N bond [54]. Further, quantitative information on the secondary structure of proteins obtained from the amide I bands are given in Figure 2C,D. The α-helix and β-sheet structures are usually regarded as the ordered structures of proteins. It could be observed that enzymatic modification significantly increased the level of α-helix and β-sheet structures in YP. Naturally, the content of ordered structures in EYP-based emulsions was higher compared to that in YP-based emulsions. After the addition of polysaccharides, the level of α-helix structures in both emulsions increased, and the EYP–CS emulsions had a 25.3% higher content of α-helical structures than EYP–XG emulsions possibly due to a stronger intermolecular interactions of EYP with CS. Alternatively, the addition of MPs reduced the level α-helix and β-turn structures as well as increased the level β-sheet structures in complex Pickering emulsions on account of the interactions of MPs with emulsions, suggesting that the binding and rearrangement of secondary structures in emulsions led to the formation of a more flexible and ductile structure [55,56]. Taken together, enzymatic modification plus polysaccharide treatment greatly promoted the formation of ordered structures in proteins and enhanced the stability of emulsions.

The 830 cm^−1^ and 850 cm^−1^ peaks in the Raman spectra of proteins were associated with the vibrations of the para-substituted benzene of tyrosine residues. The intensity ratio of Fermi bimodal (I_850_/I_830_) could be used to characterize the microenvironment of tyrosine residues and the nature of hydrogen bonds formed by the tyrosine phenolic hydroxyl groups [57]. When the I_850_/I_830_ ratio is between 0.9 and 2.5, it indicates that tyrosine residues were exposed to polar microenvironments or that the residues were both hydrogen bond acceptors and hydrogen bond donors [58]. By contrast, when the ratio was lower than 0.9, it could be inferred that the tyrosine residues were buried in hydrophobic environments or that they only acted as hydrogen bond donors to strengthen hydrogen bonds [54]. As shown in Table 2, the I850/830 ratios of YP–CS and EYP–CS as well as YP–XG and EYP–XG emulsions were in the range of 1.234–2.168, suggesting that the tyrosine residues of proteins were involved in the formation of moderate or weak hydrogen bonds. Additionally, the I_850_/I_830_ ratio of CS-based emulsions was significantly higher than that of XG-based emulsions. It is possible that a higher content of tyrosine residues in CS-based emulsions were exposed in comparison with XG-based emulsions, and the hydrogen bonding interactions of proteins with CS were much stronger than those with XG. Notably, the addition of MPs significantly reduced the I850/830 ratio in emulsions due to the interactions between MPs and the protein–polysaccharide complexes, which enabled tyrosine residues to be buried in the hydrophobic environment and led to the reduction of hydrophilicity. Notably, the MP-loaded EYP-based emulsions encapsulated with MPs had a relatively higher I_850_/I_830_ ratio than MP-loaded YP-based emulsions, indicating an increased level of tyrosine residues involving the hydrogen bonding interactions during the encapsulation of MP. In particular, both the α-helix and β-sheet structures are stabilized by hydrogen bonding [54]. Consequently, our results on the I_850_/I_830_ ratio confirmed the data of the α-helix and β-sheet structure contents in emulsions as well.

Moreover, the ratio of the normalized intensity value of the Raman spectrum at 760 cm^−1^ and 1003 cm^−1^ (I_760_/I_1003_) could reflect the hydrophobic interactions between molecules in emulsions [59]. Table 2 shows that the I_760_/I_1003_ ratio of XG-based emulsions was significantly higher than that of CS-based emulsions, suggesting stronger hydrophobic interactions between biomolecules in XG-based emulsions. XG exhibited a three-dimensional network structure in aqueous solution, which could improve the network structure of protein emulsions by enhancing the hydrophobic interactions.

### 3.8. CC Analysis of MP-Loaded Complex Pickering Emulsions

**Figure 3:**
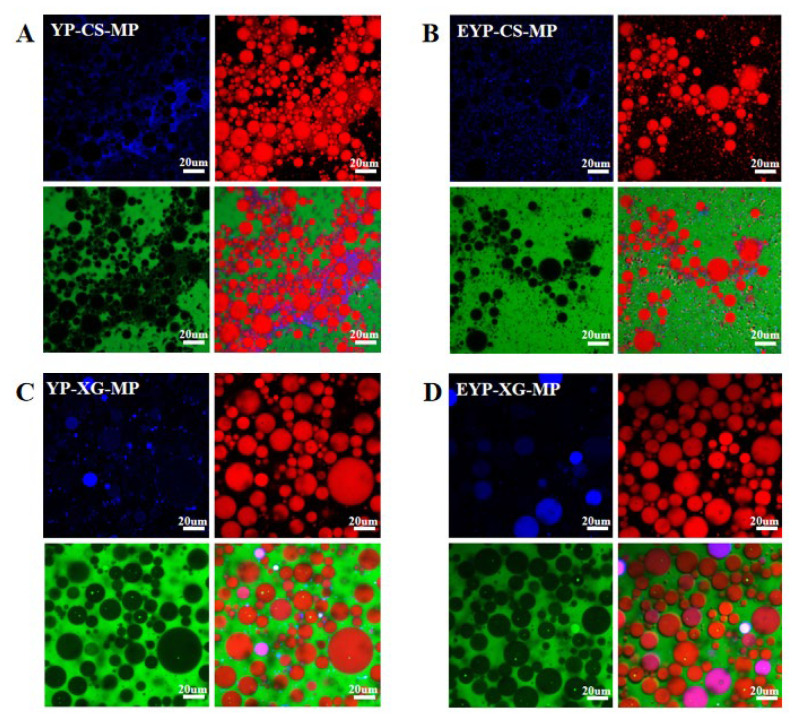
CLSM analysis of MP-loaded YP–CS (**A**), EYP–CS (**B**), YP–XG (**C**), and EYP–XG (**D**) complex Pickering emulsions at 40× magnification.

For confocal laser scanning microscopy analysis, YP and EYP molecules were stained blue using Nile blue, while oil droplets were stained red using Nile red. The aqueous phase was stained green with FITC, and the purple color of samples in micrographs was due to the superimposition of blue and red colors. As shown in Figure 3, there were significant differences in the particle size distribution and aggregation state between CS-based and XG-based emulsions. In CS-based emulsions, protein molecules and oil droplets exhibited a smaller particle size and aggregated together. However, XG-based emulsions showed a regular droplet shape and clear boundaries, while the protein molecules and oil droplets were uniformly dispersed in emulsions. Meanwhile, the particle size of XG-based emulsions were significantly higher compared with that of CS-based emulsions. This was consistent with the results of particle size analysis and probably brought about by the viscous nature of XG, which prevented the disperse of oil droplets. Moreover, the protein molecules in EYP-based emulsions were more uniformly distributed than YP-based emulsions, and the size of oil droplets in EYP-based emulsions were much smaller with no obvious aggregation. It was hypothesized that the enzymatic modification could provide more electrostatic repulsion and spatial site resistance effects to disperse the oil droplets for YP [19].

### 3.9. Effects of Storage Time on the Color Changes of MP-Loaded Complex Pickering Emulsions at Room Temperature

**Figure 4:**
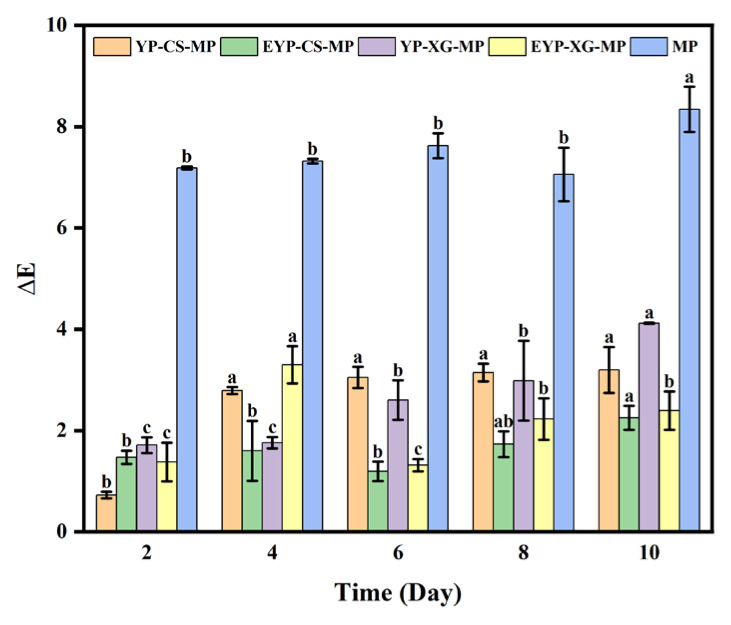
Effects of storage time on the color changes of MP-loaded complex Pickering emulsions at room temperature. Note: The ΔE value represents the color change of samples stored for a period compared with that noted at the 0th day. Where, different superscript letters indicate significant differences (*p* < 0.05).

The effects of storage time on the color changes of MP-loaded complex Pickering emulsions at room temperature are shown in Figure 4. It has been demonstrated that the chromogenic functional groups of MP molecules could absorb light energy, which led to the destruction of the molecular structure of MPs and further changed their color [4]. The ΔE value represented the color changes of different samples after storage. It could be observed that the ΔE value of all samples gradually increased with the storage time, indicating the increasing degradation degree of MPs. Whereas, the ΔE value of MP-loaded emulsions was significantly lower compare with that of control. After a storage of 10 days, the ΔE value of MP-loaded EYP–CS emulsions was only 26.99% of that of control. It could be inferred that encapsulation with emulsions significantly inhibited the degradation of MPs during storage. In addition, the ΔE value of EYP-based emulsions encapsulated with MPs was relatively lower than those of YP-based emulsions. The ΔE value of MP-loaded EYP–CS emulsions after storing for 10 days was 2.25 lower than that of MP-loaded YP–CS emulsions. In a nutshell, EYP-based emulsions might be more appropriate to be utilized in the encapsulation of MPs than YP-based emulsions. Thus, protein–polysaccharide complexation enhances emulsification and stability, rendering these emulsions effective carriers for bioactive compound delivery. Given their strong encapsulation ability and stability, these emulsions have potential applications in protecting and delivering bioactive compounds in the pharmaceutical and food industries [60].

### 3.10. The Storage Stability of MP-Loaded Complex Pickering Emulsions Under Refrigerating and Heating Conditions

**Figure 5:**
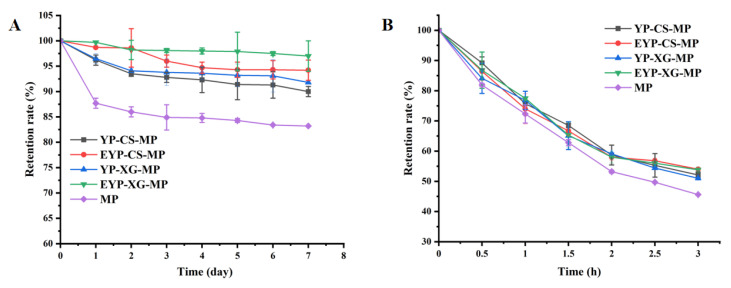
The storage stability of MP-loaded complex Pickering emulsions under refrigeration (**A**) and heating conditions (**B**). Where, different superscript letters indicate significant differences (*p* < 0.05).

To investigate the storage stability of MPs in emulsions under refrigerating and heating conditions, the retention rate of MPs in different samples after storage was evaluated. As shown in Figure 5A, the retention rate of MPs in different groups continuously decreased with storage time (0–7 days) at 4 °C. Encapsulation with emulsions remarkably increased the retention rate of MPs at 4 °C. After 7 days of storage at 4 °C, the retention rate of MPs in EYP–XG emulsions was 13.8% higher than that of control. In addition, the retention rate of MPs in EYP-based emulsions was relatively higher compared with that in YP-based emulsions. Notably, the retention rate of MPs in EYP–XG emulsions was 5.2% higher than that of YP-emulsions. It is possible that the EYP molecules adsorbed on the interfacial layer more easily interacted with CS and XG, which strengthened their protection of MPs. The effects of heating time on the stability of MPs in different emulsions were similar to those of refrigerating (Figure 5B). It could be observed that the retention rate of MPs in different groups decreased with the heating time (0–3 h), while encapsulation with emulsions effectively inhibited the degradation of MPs. After heating at 80 °C for 3 h, the retention rate of MP-loaded EYP–CS emulsions was 8.4% higher in comparison with that of control. Also, the retention rate of MPs in EYP-based emulsions after different heating times was relatively higher than that in YP-based emulsions. These results indicate that EYP-based emulsion encapsulation effectively preserved MP stability, mitigating degradation under refrigerating and heating conditions. Thus, protein–polysaccharide complexation enhances emulsification and stability, making these emulsions effective carriers for active substance delivery. Given their strong encapsulation ability and stability, these emulsions have potential applications in protecting and delivering bioactive compounds in the pharmaceutical and food industries.

## 4. Conclusions

The processing instability of *Monascus* pigments poses significant challenges in their industrial applications. Encapsulation using emulsion systems can greatly enhance their processing stability. The present study investigated the stabilizing effects of enzymatically modified yeast protein–polysaccharide composite emulsions on *Monascus* pigments. Our results demonstrated that enzymatic modification significantly enhanced the emulsifying activity of yeast proteins, primarily attributed to structural unfolding and increased exposure of hydrophobic amino acids. Protein–polysaccharide composite emulsions exhibited superior stability, with XG-based systems showing greater physical stability, while CS-based emulsions provided more effective encapsulation efficiency for the bioactive compounds. Compared to YP/EYP–XG emulsions, YP/EYP–CS emulsions had smaller average particle size and stronger hydrogen-bonding interactions. Rheological analysis confirmed shear thinning behavior, with YP/EYP–XG emulsions showing higher viscosity and elasticity. Structural analysis revealed that EYP–CS emulsions had the most ordered structure and highest α-helix content. Additionally, EYP–CS emulsions exhibited better encapsulation efficiency of MP as well as improved its color retention and storage stability. It could be inferred that combining proteins with superior emulsifying properties and high-viscosity polysaccharides could produce emulsions with enhanced stability. However, encapsulation performance requires careful consideration of their molecular interactions. Notably, emulsion-encapsulated *Monascus* pigments consistently exhibited greater stability than their free counterparts. Thus, protein–polysaccharide complexation enhances emulsification and stability, making these emulsions effective carriers for active substance delivery.

## Figures and Tables

**Table 1:** Effects of MP concentration on the encapsulation efficiency of MPs in different emulsions.

MP Concentration	Encapsulation Efficiency (%)			
	YP–CS	EYP–CS	YP–XG	EYP–XG
0.10% (*w*/*v*)	76.33 ± 0.02 ^d^	71.00 ± 0.12 ^e^	56.45 ± 0.25 ^l^	52.52 ± 1.1 ^m^
0.15% (*w*/*v*)	79.34 ± 0.89 ^b^	79.73 ± 1.01 ^b^	62.99 ± 0.4f ^g^	58.60 ± 0.3 ^k^
0.20% (*w*/*v*)	78.09 ± 0.5 ^c^	81.18 ± 0.18 ^a^	62.21 ± 0.11g ^h^	62.79 ± 0.24 ^fg^
0.25% (*w*/*v*)	78.97 ± 0.07 ^b^	79.20 ± 0.7 ^b^	63.39 ± 0.19 ^f^	61.03 ± 0.49 ^i^
0.30% (*w*/*v*)	76.56 ± 0.91 ^d^	76.18 ± 0.02 ^d^	61.36 ± 0.52 ^hi^	59.66 ± 0.05 ^g^

where, different superscript letters indicate significant differences (*p* < 0.05).

**Table 2:** The tan*δ* values, normalized intensity of 760 cm^−1^ bands, and ratio I_850_/I_830_ doublet bands of different emulsions.

Sample	tan*δ*	I_850_/I_830_	I_760_/I_1003_
YP–CS	0.23 ± 0.00 ^ef^	2.06 ± 0.01 ^a^	0.37 ± 0.06 ^d^
EYP–CS	0.25 ± 0.04 ^de^	1.98 ± 0.06 ^a^	0.43 ± 0.06 ^d^
YP–CS–MP	0.20 ± 0.01 ^f^	0.56 ± 0.09 ^c^	1.29 ± 0.03 ^c^
EYP–CS–MP	0.88 ± 0.05 ^a^	1.18 ± 0.10 ^b^	1.02 ± 0.09 ^c^
YP–XG	0.29 ± 0.00 ^cd^	1.23 ± 0.03 ^b^	3.66 ± 0.08 ^a^
EYP–XG	0.28 ± 0.00 ^cd^	1.34 ± 0.14 ^b^	1.92 ± 0.03 ^b^
YP–XG–MP	0.36 ± 0.00 ^b^	0.22 ± 0.08 ^d^	1.37 ± 0.07 ^c^
EYP–XG–MP	0.29 ± 0.04 ^c^	0.44 ± 0.02 ^cd^	1.74 ± 0.13 ^b^

where, different superscript letters indicate significant differences (*p* < 0.05).

## Data Availability

The original contributions presented in this study are included in the article/supplementary materials. Further inquiries can be directed to the corresponding author(s).

## Supplementary Materials

The following supporting information can be downloaded at: https://www.mdpi.com/article/10.3390/foods14081366/s1, Figure S1: Effects of different enzyme treatments on the degree of hydrolysis (DH), solubility, emulsifying activity index (EAI), and emulsifying stability index (ESI) of YP. Note: P: Papain-treated group, P-TG: Papain plus TG-treated group, A: Alkaline protease-treated group, A-TG: Alkaline protease plus TG-treated group, N: Neutral protease-treated group, N-TG: Neutral protease plus TG-treated group, TG: TG-treated group. Data are expressed as mean ± SD (n = 3), repeated measures one-way ANOVA followed by DMRT. Data marked with the same letter exhibited no significant difference at *p* < 0.05; Figure S2: Effects of the additive amount of papain (A), pH value (B), reaction temperature (C), and reaction time (D) on the emulsifying activity index (EAI, bar plots) and emulsion stability index (ESI, line plots) of YP.; Figure S3:Effects of the additive amount of TG (A), pH value (B), reaction temperature (C), and reaction time (D) on the emulsifying activity index (EAI, bar plots) and emulsion stability index (ESI, line plots) of YP; Figure S4: UV spectra of YP and EYP; Figure S5: Intrinsic fluorescence spectra of YP and EYP; Figure S6: Particle size distribution of YP and EYP; Figure S7: Fourier transform infrared spectroscopy of YP and EYP; Figure S8: Electrophoresis patterns of YP and EYP; Figure S9: Scanning electron microscopy images of YP (A,B) and EYP (C,D) at 3000× magnification (left) and 6000× magnification (right); Figure S10: Effects of pH on the particle size (A,D), zeta potential (B,E), emulsifying activity index (EAI, bar plots) (C,F), and emulsion stability index (ESI, line plots) of CS-based and XG-based complex Pickering emulsions; Figure S11: Effects of protein/polysaccharide ratio on the particle size (A,D), zeta potential (B,E), emulsifying activity index (EAI, bar plots) (C,F), and emulsion stability index (ESI, line plots) of CS-based and XG-based complex Pickering emulsions; Figure S12: Effects of the additive amount of oil on the particle size (A,D), zeta potential (B,E), emulsifying activity index (EAI, bar plots) (C,F), and emulsion stability index (ESI, line plots) of CS-based and XG-based complex Pickering emulsions; Table S1: Secondary structure of YP and EYP peptide; Table S2: Molecular weight distribution of YP and EYP; Table S3: Effects of pH, protein/polysaccharide ratio, and the additive amount of oil on the creaming index (CI) of CS-based and XG-based complex Pickering emulsions.
