# Supplementary material for: Encapsulation of Monascus Pigments Using Enzyme-Modified Yeast Protein–Polysaccharide Complex Pickering Emulsions to Increase Its Stability During Storage
Source: Foods. 2025 Apr 15;14(8):1366. doi: 10.3390/foods14081366 (PMC12027061; doi:10.3390/foods14081366)
Supplement: Supplementary file 1 [file foods-14-01366-s001.zip › foods-3519256-supplementary.pdf]

# Supplementary Materials

## Encapsulation of *Monascus* Pigments using Enzyme-Modified Yeast Protein–Polysaccharide Complex Pickering Emulsions to Increase Its Stability During Storage


Ziyan Zhao[1, 2, 3], Jinling Zhao[1, 2, 3], Sirong Liu[1, 2, 3], Mengxuan Liu[1, 2, 3], Xiangquan Zeng[1, 2, 3*] He Li[1, 2, 3], Yu Xi[1, 2, 3], Jian Li[1, 2, 3*]

(1 Beijing Technology and Business University, Key Laboratory of Geriatric Nutrition and Health, Ministry of Education, Beijing, 100048, PR China; 2 Key Laboratory of Green and Low-carbon Processing Technology for Plant-based Food of China National Light Industry Council, School of Food and Health, Beijing Technology and Business University, Beijing, 100048, PR China; 3 Beijing Engineering and Technology Research Center of Food Additives, School of Food and Health, Beijing Technology and Business University, Beijing, 100048, PR China)

**Corresponding Author**

Xiangquan Zeng, E-mail: 20210803@btbu.edu.cn

Jian Li, E-mail: lijian@th.btbu.edu.cn

**Authors**

Ziyan Zhao, E-mail: 18726926317@163.com

Jinling Zhao, E-mail: 15110631956@163.com

Sirong Liu, E-mail: 15223376402@163.com

Mengxuan Liu, E-mail: 15545957141@163.com

He Li, E-mail: lihe@btbu.edu.cn

Yu Xi, E-mail: xiyu@btbu.edu.cn

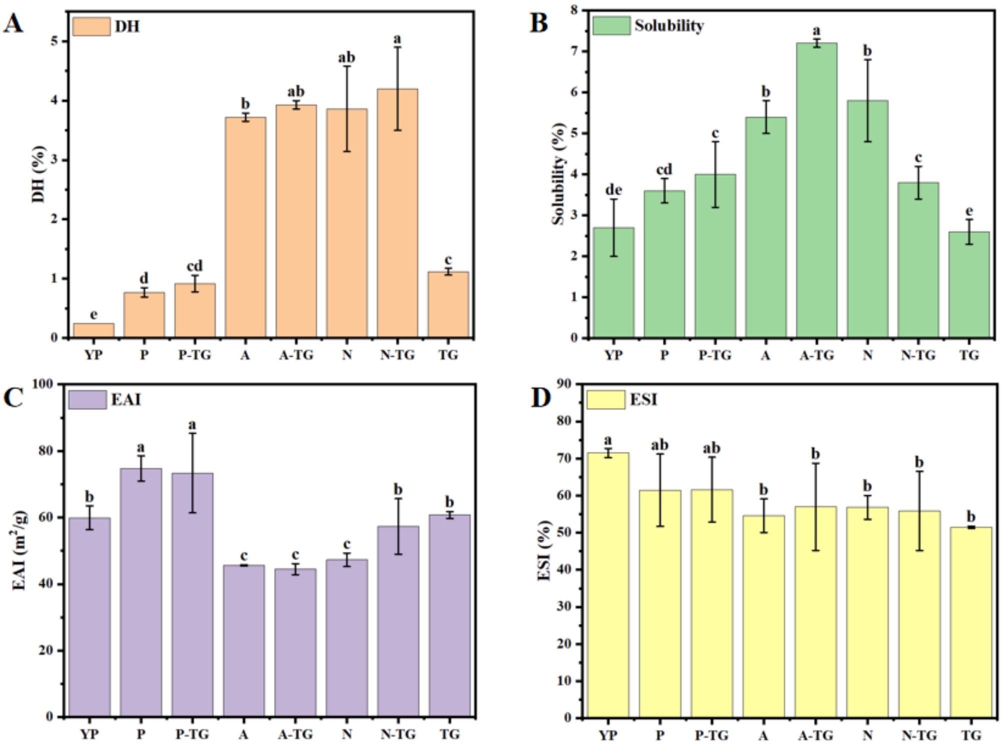


**Fig. S1.** Effects of different enzyme treatments on the degree of hydrolysis (DH), solubility, emulsifying activity index (EAI), and emulsifying stability index (ESI) of YP. Note: P: Papain-treated group, P-TG: Papain plus TG-treated group, A: Alkaline protease-treated group, A-TG: Alkaline protease plus TG-treated group, N: Neutral protease-treated group, N-TG: Neutral protease plus TG-treated group, TG: TG-treated group. Data are expressed as mean ± SD (n = 3), repeated measures one-way ANOVA followed by DMRT. Data marked with the same letter exhibited no significant difference at $p < 0.05$.

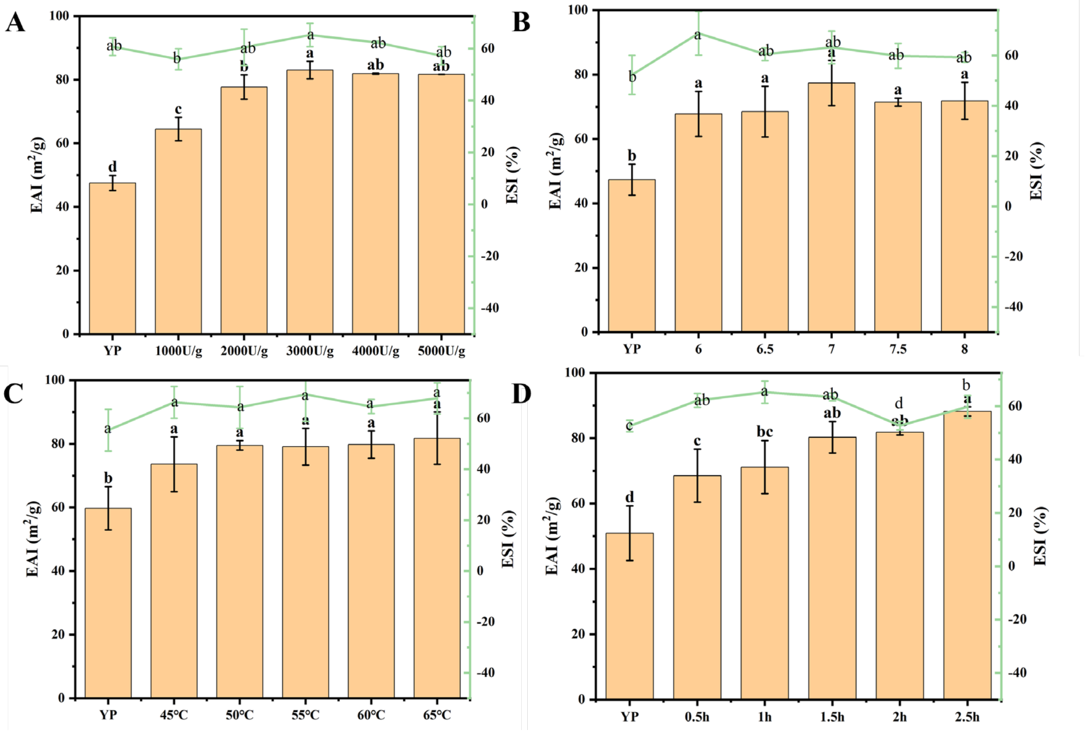


**Fig. S2.** Effects of the additive amount of papain (A), pH value (B), reaction temperature (C), and reaction time (D) on the emulsifying activity index (EAI, bar plots) and emulsion stability index (ESI, line plots) of YP.

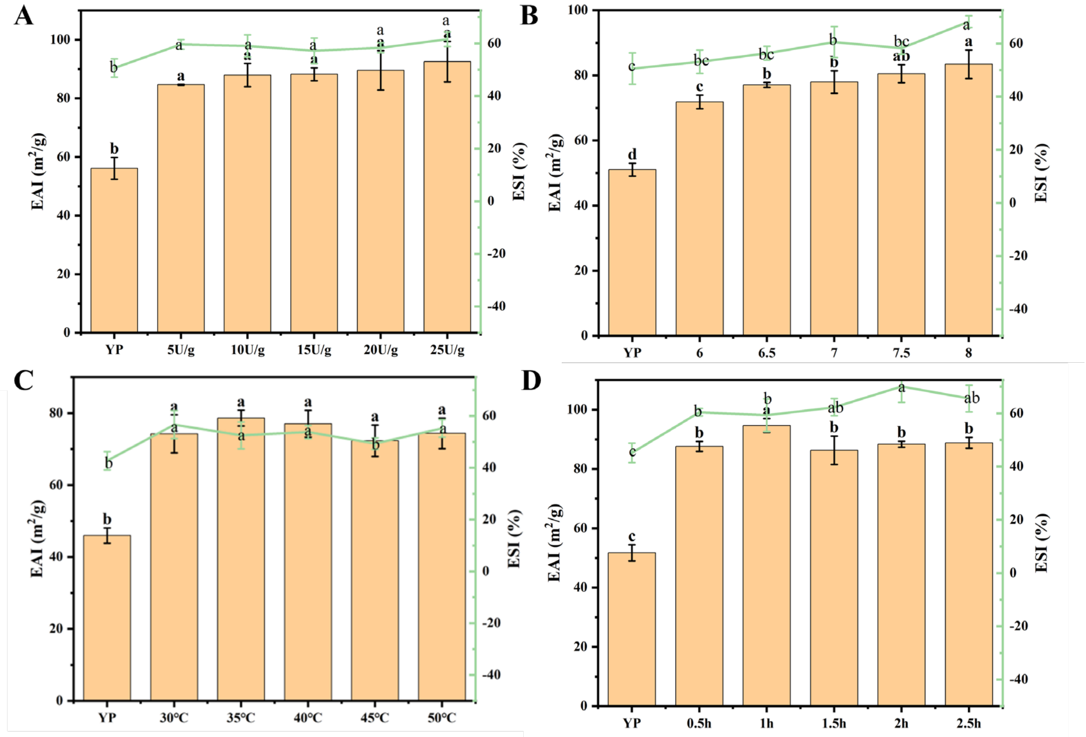


**Fig. S3.** Effects of the additive amount of TG (A), pH value (B), reaction temperature (C), and reaction time (D) on the emulsifying activity index (EAI, bar plots ) and emulsion stability index (ESI, line plots ) of YP.

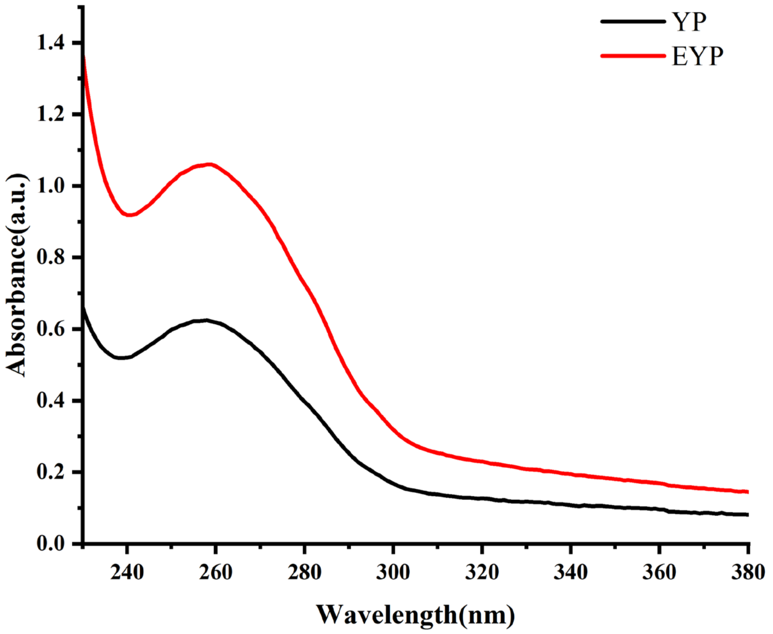


**Fig. S4.** UV spectra of YP and EYP.

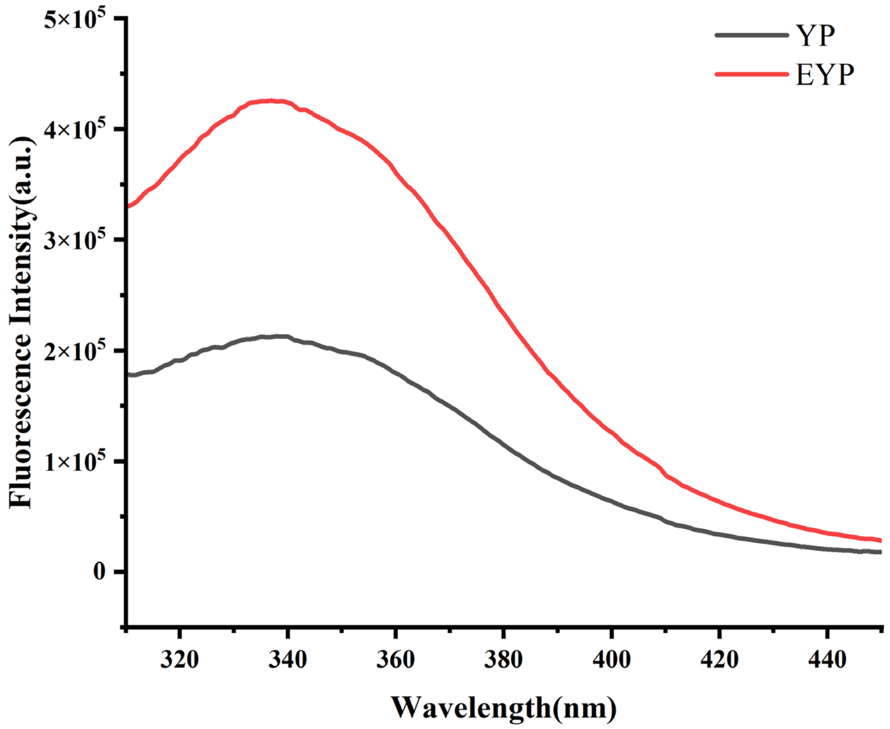


**Fig. S5.** Intrinsic fluorescence spectra of YP and EYP.

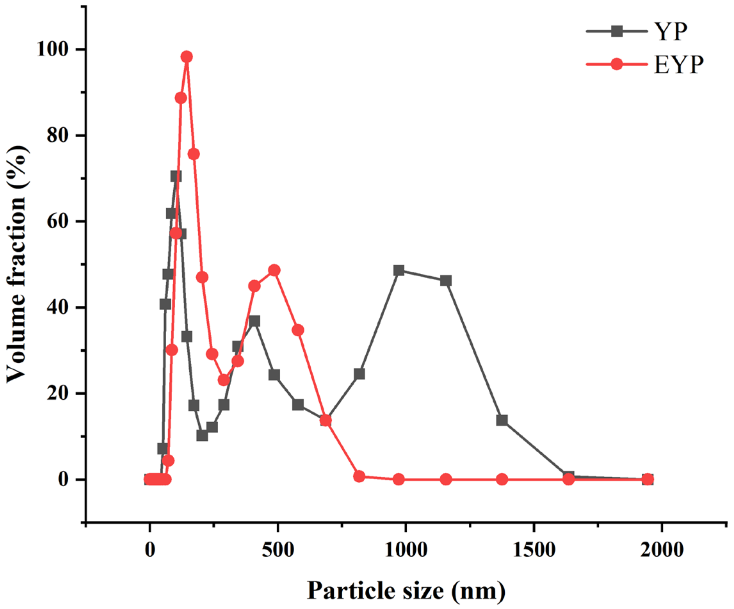


**Fig. S6.** Particle size distribution of YP and EYP.

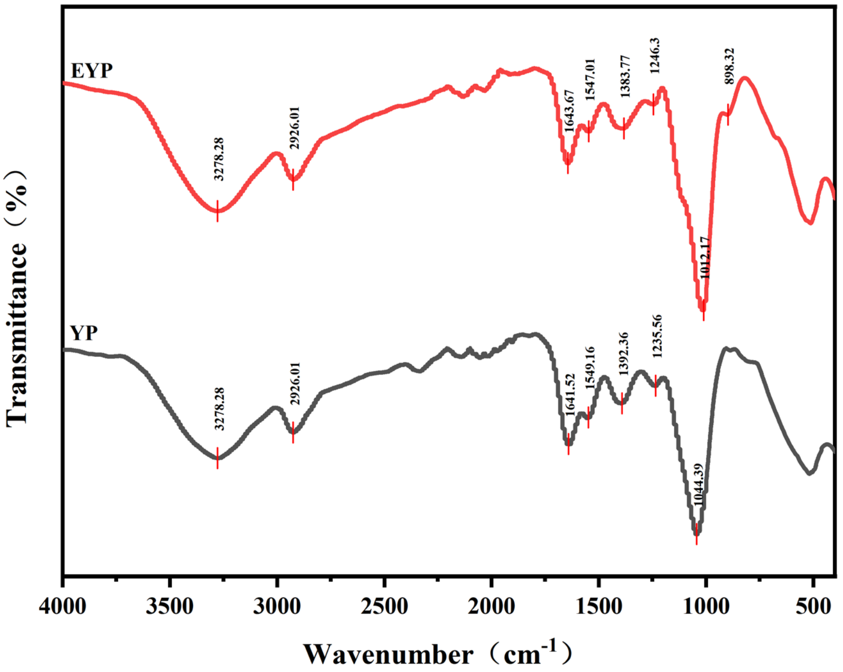


**Fig. S7.** Fourier transform infrared spectroscopy of YP and EYP.

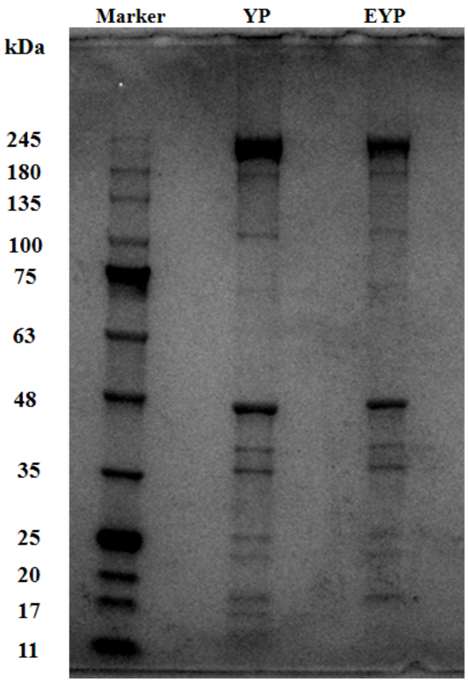


**Fig. S8.** Electrophoresis patterns of YP and EYP.

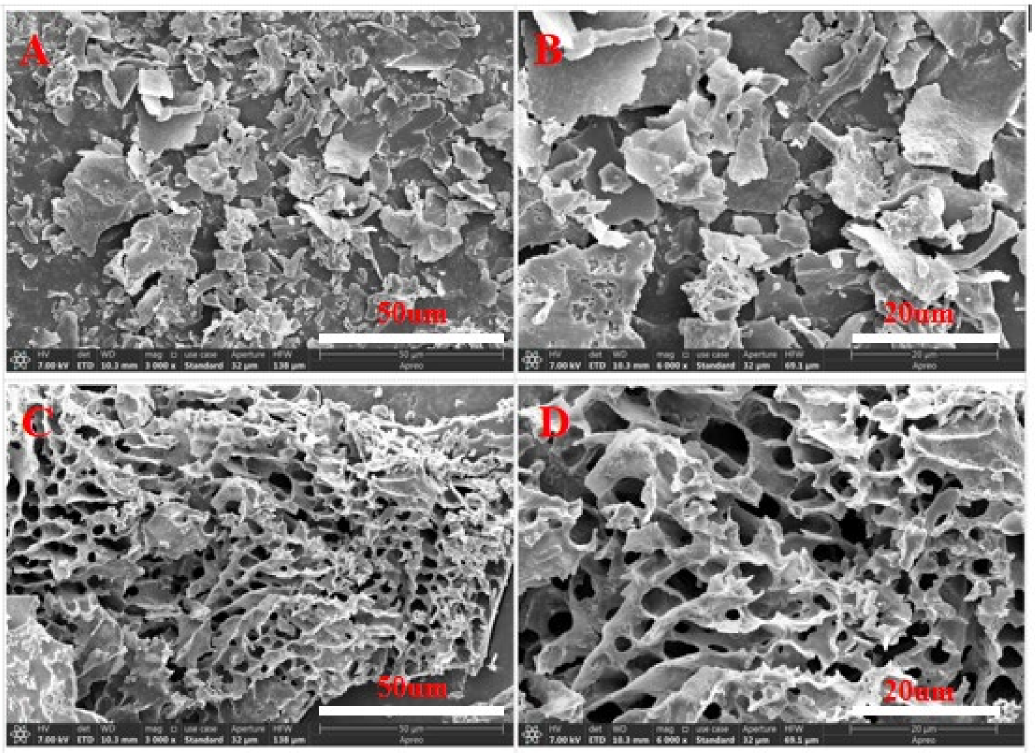


**Fig. S9.** Scanning electron microscopy images of YP (A , B) and EYP (C, D) at 3000× magnification (left) and 6000× magnification (right).

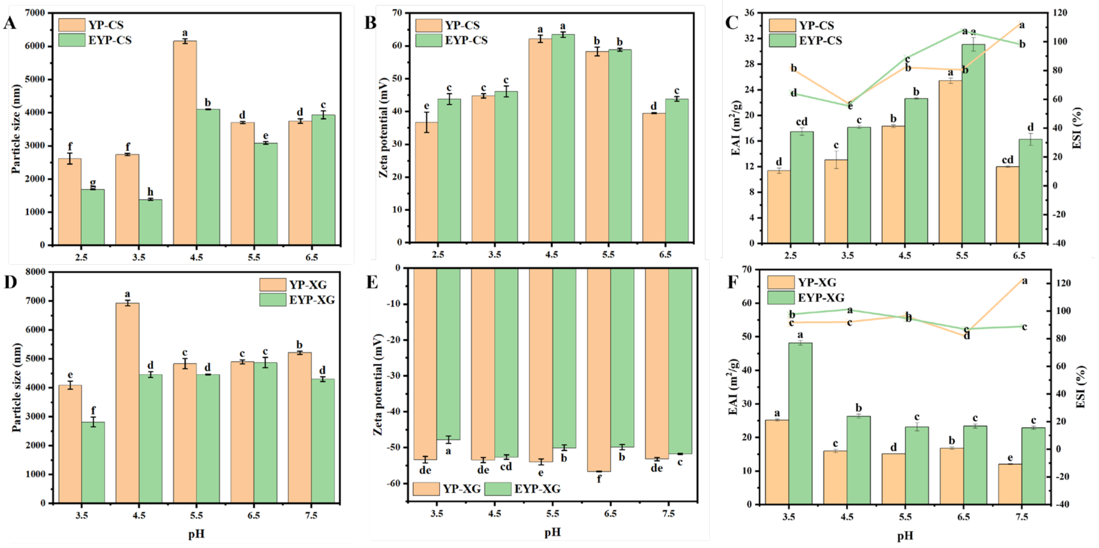


**Fig. S10.** Effects of pH on the particle size (A, D), zeta potential (B, E), emulsifying activity index (EAI, bar plots) (C, F), and emulsion stability index (ESI, line plots) of CS-based and XG-based complex Pickering emulsions.

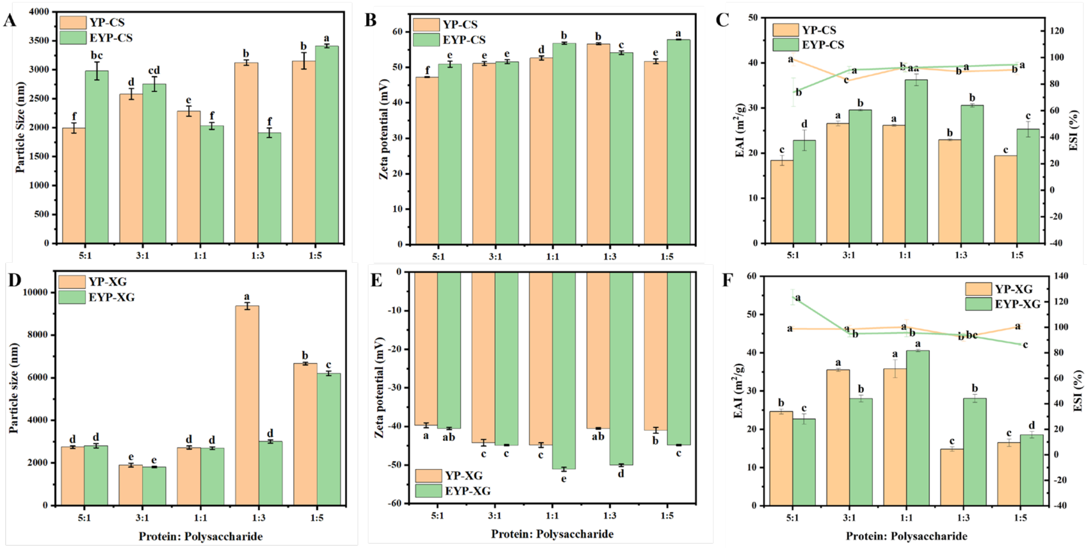


**Fig. S11.** Effects of protein/polysaccharide ratio on the particle size (A, D), zeta potential (B, E), emulsifying activity index (EAI, bar plots) (C, F), and emulsion stability index (ESI, line plots) of CS-based and XG-based complex Pickering emulsions.

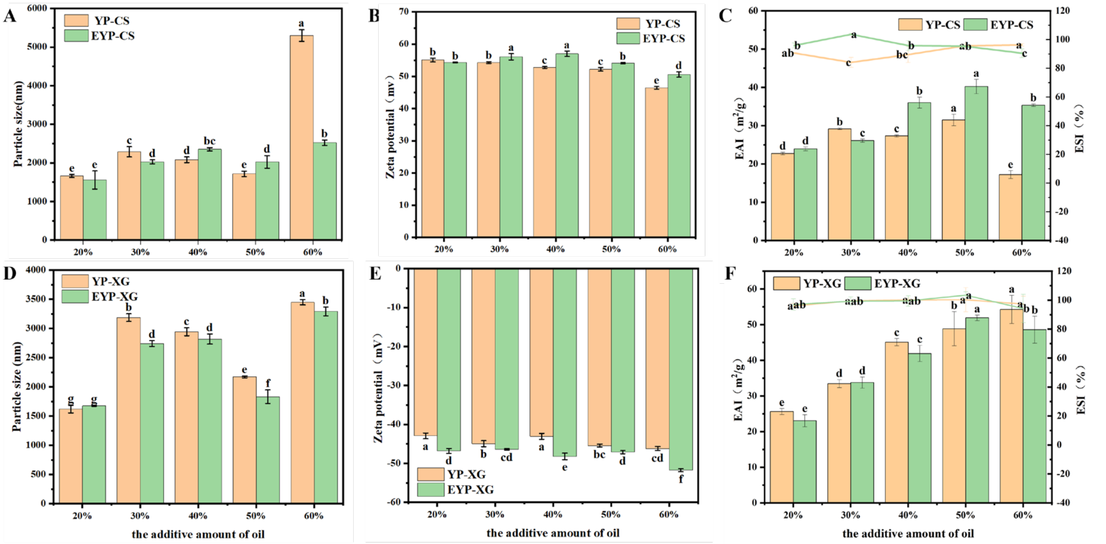


**Figure S12.** Effects of the additive amount of oil on the particle size (A, D), zeta potential (B, E), emulsifying activity index (EAI, bar plots) (C, F), and emulsion stability index (ESI, line plots) of CS-based and XG-based complex Pickering emulsions.

**Table S1.** Secondary structure of YP and EYP peptide.

| Samples | β-sheet （%） | random coil （%） | α-helix （%） | β-turn （%） |
|---|---|---|---|---|
| YP | 36.47±0.2[a] | 14.57±0.43[d] | 20.09±0.03[c] | 28.87±0.26[b] |
| EYP | 32.48±1.26[a] | 14.30±0.20[c] | 20.79±0.66[b] | 32.43±0.40[a] |

**Table S2.** Molecular weight distribution of YP and EYP.

| **Sample** | **Mn** | **Mw** | **Mz** | **PDI** |
|---|---|---|---|---|
| YP | $4.68\times10^{3}$ | $5.03\times10^{5}$ | $1.11\times10^{7}$ | $1.07\times10^{2}$ |
| EYP | $3.04\times10^{3}$ | $1.79\times10^{5}$ | $8.86\times10^{6}$ | $5.88\times10$ |

**Table S3.** Effects of pH, protein/polysaccharide ratio, and the additive amount of oil on the creaming index (CI) of CS-based and XG-based complex Pickering emulsions.

| Group | CI（%） | | | | | |
|---|---|---|---|---|---|---|
| | Variable | YP–CS | EYP–CS | Variable | YP–XG | EYP–XG |
| pH | 2.5 | 48.42±0.10$^{g}$ | 50.53±0.04$^{f}$ | 3.5 | 87±0.22$^{d}$ | 87.82±0.1$^{b}$ |
| | 3.5 | 50.53±0.11$^{f}$ | 52.21±0.18$^{e}$ | 4.5 | 86.6±0.02$^{e}$ | 87.65±0.04$^{bc}$ |
| | 4.5 | 52.63±0.12$^{d}$ | stratified | 5.5 | 87.37±0.07$^{c}$ | 88.21±0.11$^{a}$ |
| | 5.5 | 58.95±0.11$^{c}$ | stratified | 6.5 | 85.26±0.15$^{f}$ | 61.05±0.09$^{h}$ |
| | 6.5 | 80±0.27$^{b}$ | 88.42±0.11$^{a}$ | 7.5 | 87.37±0.16$^{c}$ | 65.26±0.43$^{g}$ |
| Protein: polysaccharide | 5:1 | 56.84±0.13$^{d}$ | 59.06±0.03$^{b}$ | 5:1 | 87.2±0.33$^{g}$ | 88±0.32$^{f}$ |
| | 3:1 | 56.39±0.05$^{e}$ | 58.59±0.31$^{c}$ | 3:1 | 91.2±0.12$^{e}$ | 92±0.45$^{d}$ |
| | 1:1 | 59.79±0.06$^{a}$ | 59.21±0.09$^{b}$ | 1:1 | 96±0.33$^{c}$ | 98±0.7$^{b}$ |
| | 1:3 | stratified | stratified | 1:3 | 98±0.3$^{b}$ | 99.6±0.1$^{a}$ |
| | 1:5 | stratified | stratified | 1:5 | 99.6±0.03$^{a}$ | stable |
| The additive amount of oil | 20% | stratified | stratified | 20% | 87.2±0.18$^{e}$ | 88±0.21$^{e}$ |
| | 30% | stratified | stratified | 30% | 91.2±0.8$^{d}$ | 92±0.66$^{d}$ |
| | 40% | stratified | stratified | 40% | 96±0.33$^{c}$ | 98±0.45$^{b}$ |
| | 50% | 78.63±0.08$^{d}$ | 81.58±0.2$^{b}$ | 50% | stable | 99.6±0.2$^{a}$ |
| | 60% | 80.29±0.17$^{c}$ | 90.65±0.63$^{a}$ | 60% | stable | stable |

YP/EYP–CS and YP/EYP–XG were separated for significant analysis pH, protein:polysaccharide and the additive amount of oil.
